# Numerical modeling of the dissolution of drug nanocrystals and its application to industrial product development^1^

**DOI:** 10.5599/admet.1437

**Published:** 2022-08-04

**Authors:** Bastian Bonhoeffer, Andreas Kordikowski, Edgar John, Michael Juhnke

**Affiliations:** Novartis Pharma AG, Technical R&D, Forum 1, Novartis Campus, 4056 Basel, Switzerland

**Keywords:** Bioavailability, drug particles, enabling formulation, nanosuspension, supersaturation, wet media milling

## Abstract

The apparent solubility of drug nanocrystals in equilibrium was experimentally determined for a drug-stabilizer system with different particle size distributions. True supersaturation was identified for ultrafine drug nanocrystals with an almost 2-fold increase compared to the thermodynamic solubility of related coarse drug crystals, highlighting their enabling potential to enhance bioavailability. The experimental results were applied to investigate *in silico* the associated dissolution behavior in a closed system by numerical modeling according to the Ostwald-Freundlich and Noyes-Whitney / Nernst-Brunner equations. Calculated results were found to be in agreement with the experimental results only when the entire particle size distribution of drug nanocrystals was considered. *In silico* dissolution, studies were conducted to simulate the complex interplay between drug nanocrystals, dissolution conditions and resulting temporal progression during dissolution up to the equilibrium state. Calculations were performed for selected *in vivo* and *in vitro* scenarios considering different drug nanocrystal particle size distributions, drug amount, dissolution media and volume. The achieved results demonstrated the importance of ultrafine drug nanocrystals for potential bioavailability improvement and the functional applicability of the modeling approach to investigate their dissolution behavior for configurable formulation variables in product development in terms of *in vivo* and *in vitro* relevant conditions.

## Introduction

In the last three decades, nanocrystals have emerged as a formulation strategy to improve bioavailability-related problems of poorly-soluble drugs, enhance clinical convenience, and enable specific therapeutic benefits [1-7]. Until now, several commercialized nanocrystalline drug products reached the market for oral, ocular and injection (sc, im, iv) routes of administration [8-10]. Beyond that, a small number of nanocrystalline drug products are continuously approaching early development and the generic product lifecycle [9]. For oral products, the improvement of the drug dissolution rate through an increase of the drug nanocrystal specific surface area, the increase of the solubility due to ultrafine nanocrystals and the adhesion of nanocrystals to the gut wall are considered the main contributing factors to bioavailability improvement [8,11]. On the other hand, ocular and injectable products are most often associated with improved clinical convenience, like the ease of administration and reduced dosing frequency or specific therapeutic benefits like continuous and controlled release [12-14].

The manufacturing of drug nanocrystals for industrial applications is most often performed by wet media milling technology [15-19]. In essence, size reduction of drug particles takes place in aqueous suspension in the presence of stabilizers between colliding grinding media. The technology is in a mature state and considered a versatile drug delivery platform owing to its industrial applicability for oral, ocular, and injectable products from the pre-clinical up to the commercial stage. Wet media milled drug nanocrystals are mostly reported with product particle sizes between about 150 to 300 nm. Interestingly, only a few reports are available on the manufacturing of sub-100 nm drug nanocrystals through an optimized design of the wet media milling process [20,21].

The underlying mechanisms for bioavailability improvement of marketed oral nanocrystal products were investigated by *in vitro*/*in silico* endeavors to retrospectively understand *in vivo* absorption [11,22-30]. There is a general agreement that the increase of the drug nanocrystal-specific surface area is a contributing factor for the dissolution rate and the corresponding bioavailability improvement. Further contributing factors for bioavailability improvement cannot always be fully explained and hence cannot be generalized. Hypothesized additional factors include, *i.e.*, intestinal absorption of intact nanocrystals into the enterocytes, increased drug concentration at the epithelial surface, increased deposition and retention of nanocrystals due to mucoadhesion and positive effects of surfactants on drug solubility [11,22,24-28]. Supersaturation from ultrafine nanocrystals is not considered a contributing factor to bioavailability improvement. However, there is no compelling *in vivo* evidence to directly validate or invalidate any of these further mechanisms.

Dissolution kinetics and dissolution-permeation tests are typical approaches to investigate *in vitro* the contributing factors for the potential bioavailability improvement of oral nanocrystal products [31-41]. In general, the dissolution kinetics of dispersed nanocrystals takes place almost instantaneously, *i.e.*, in the time scale of seconds. Therefore, the discrimination of the dissolution rate of dispersed nanocrystals with different particle sizes, even in comparison to dispersed microcrystals, is challenging. Furthermore, the separation of undissolved nanocrystals from the dissolved drug for the determination of the dissolved mass fraction is considered difficult and reported data in the literature must be critically reviewed [8]. *In situ* approaches using, *i.e.*, ion selective electrodes, second-derivative UV spectroscopy and dynamic light scattering could be a possibility [29,35,40,41]. Although it must be borne in mind that the interaction of UV-radiation with ultrafine nanocrystals and the poor time resolution for UV spectroscopy-based methods are still challenging [42]. Dissolution-permeation tests were also evaluated overcoming the issue of separation of dissolved from the undissolved drug when relying on data obtained from the acceptor compartment [23,27,29,32-39]. These tests provide an overall, interrelated set of information without discrimination between dissolution and permeation. Case studies provided a rank ordering for different formulations with often promising correlation to the related *in vivo* absorption data. However, the appropriate experimental set-up and parameters of the permeation test, including membrane properties, donor and acceptor media, volume and hydrodynamics, should be considered [37,39].

Supersaturation, respectively the increase of apparent solubility in relation to the thermodynamic solubility due to nanocrystal particle size, as described by the Ostwald-Freundlich equation, is another potential contributing factor to the bioavailability improvement of oral nanocrystal products [8,43-46]. Initial studies suggested a solubility increase due to nanocrystal particle size from marginal up to several folds. The critical review of the available reports provided evidence that the increase of the apparent solubility, in relation to the thermodynamic solubility, is only marginally of up to about 15 to 20 % for the finest mean nanocrystal particle size of about 150 nm [8,43-46]. The reason for the discrepancy in the available reports was mainly associated with inadequate methods for the separation of dissolved drug from undissolved drug nanocrystals [8]. In conclusion, there is the general agreement that dissolution rate is a contributing factor and supersaturation is of secondary importance for bioavailability improvement. The bioavailability improvement due to the projected increase of the apparent solubility by the Ostwald-Freundlich equation is hypothesized to be more pronounced only for nanocrystal particle sizes in the 50 nm range [8].

Dissolution modeling is a well-established *in silico* approach to mathematically describe the diffusion-limited release from drug particles for coarse and micron particle sizes considering the complex interplay of relevant parameters such as drug amount, particle size, shape, as well as dissolution media and related volume [47-52]. In contrast, dissolution modeling from drug nanocrystals is reported only by a limited number of studies [30,53-55]. Liu et al. simulated the dissolution of monodisperse particles with a size of 1300, 560 and 340 nm, not considering the increase of the apparent solubility [30]. Ely et al. provided the theoretical framework to estimate the dissolution kinetics of monodisperse nanocrystals considering the increase of the apparent solubility by the Ostwald-Freundlich equation with the assumption of a constant diffusion boundary layer thickness [53]. Johnson simulated the dissolution of polydisperse particle size distributions considering the increase of the apparent solubility according to the Ostwald-Freundlich equation [54]. He provided simulation results for the dissolution of coarse and micron-sized particle size distributions and used experimental dissolution data for model fitting, taking the diffusion boundary layer thickness as a fit parameter. Parks et al. presented a molecular dynamics-based simulation methodology on an atomistic level and showed results for the dissolution of nanocrystals with particle sizes below 6 nm [55].

The objective of this study was to manufacture a wide range of nanocrystal particle sizes by wet media milling technology with identical drug compound and formulation composition, including sub-100 nm particle sizes using an industrially applicable process design. The manufactured drug nanocrystals were characterized for their product attributes, namely particle size distribution, morphology and solid-state properties. Subsequently, the different batches were experimentally characterized for their apparent solubility in different dissolution media. The experimental data set comprising different drug nanocrystal particle size distributions and their related apparent solubility were used to develop a numerical model for the *in silico* calculation of the dissolution kinetics of drug nanocrystals according to the Noyes-Whitney / Nernst-Brunner and Ostwald-Freundlich equations. The numerical model was then applied to selected industrially relevant *in vivo* and *in vitro* scenarios to predict *in silico* the dissolution in a closed system considering the interplay between input parameters: drug nanocrystal particle size distribution, drug amount, dissolution media and related volume, and output parameters: apparent solubility, remaining drug particle size distribution if any and drug fraction dissolved, all time-resolved up to equilibrium condition.

## Experimental

### Materials

A proprietary crystalline drug provided by Novartis Pharma AG was used for this study. The compound is a weak base showing high permeability and low solubility at physiological pH with a molecular weight above 500 g/mol, a melting point above 180 °C and water solubility below 0.1 mg/mL. A typical polymer and a surfactant are used for the stabilization of the nanocrystal suspension, according to the literature [15-19]. Purified water was used throughout all experiments as the continuous phase for nanocrystal manufacturing.

### Experimental methods

Drug nanocrystals were manufactured in aqueous suspension using wet media milling technology in recirculation mode. The drug and stabilizer composition were constant throughout the experiments, with concentrations of 25 %w drug, 4 %w stabilizers and 71 %w purified water. Nanocrystals with a target particle size > 100 nm were manufactured with the wet media mill Labstar, Netzsch Feinmahltechnik using grinding media made from yttrium stabilized zirconia with a diameter of 100 or 300 μm. Nanocrystals with a target particle size < 100 nm were manufactured with the wet media mill Ultra Apex Mill 015, Hiroshima Metal & Machinery, using grinding media made from yttrium stabilized zirconia with a diameter of 30 μm. The milling operations were performed with a rotor tip speed of 12 or 14 m/s and a duration of up to 8 hours. The manufacturing of the nanocrystals was controlled by an appropriate cooling installation to ensure maximum temperatures of the suspension between 20 to 25 °C.

The surface energy of the drug compound was characterized by drop shape analysis of the static contact angle using the drop shape analyzer DSA100, Krüss, and as complementary technique inverse gas chromatography, using the modular equipment NeuronIC, Adscientis, which includes the gas chromatograph Clarus 580, PerkinElmer. Surface energy by drop shape analysis was performed with diiodomethane and water to characterize the polar and disperse part of the surface energy using the Owens-Wendt-Rabel-Kaelble (OWRK) method for data evaluation [56,57]. Surface energy by inverse gas chromatography was performed at infinite dilution with 15 gas probes, including n-alkanes, cyclic and branched alkanes and polar gases, to characterize the disperse part of the surface energy using the Dorris-Gray method for data evaluation [58].

Particle size was characterized for the coarse crystal suspension by laser light diffraction (LLD) and for wet media milled nanocrystal suspension samples by photon correlation spectroscopy (PCS). LLD analysis was performed using the equipment model Sucell/Helos, Sympatec with the measuring range R2 by dispersing the powder in purified water with minute amounts of the dispersing aid (10 %w Tween 20) and sonication of the test dispersion until the primary particle size distribution was reached. The obtained angular scattered light was evaluated according to Fraunhofer light diffraction theory and results were reported by volume-based (*Q*_3_) particle size distribution [59]. PCS analysis was performed using the equipment model Zetasizer Nano ZS, Malvern Instruments, by dilution of the nanocrystal suspension in sterile filtered 0.1 mM NaCl aqueous solution. The obtained scattered light intensity dynamics was evaluated according to the cumulant method and results were reported by scattering intensity weighted mean particle size and polydispersity index as a measure of the width of the particle size distribution [60]. Further, the refractive index of the drug compound was determined according to the Becke line phenomenon [61]. The determined refractive index was used as an input parameter to calculate the volume-based (Q_3_) particle size distribution from the scattering intensity weighted mean particle size and polydispersity index of the PCS analysis using the built-in instrument software. As a complementary analysis, particle size and morphology of nanocrystals were characterized by scanning electron microscopy (SEM) using the equipment model GeminiSEM 300, Carl Zeiss Microscopy. All images were obtained using an accelerating voltage of 5 kV. Sample preparation was performed by filtration of the nanocrystal suspension through a nucleopore membrane filter and sputter-coated with gold.

The saturation solubility of nanocrystals was determined by the addition of the required amount of nanocrystal suspension with known assay into 1 L of either 0.01 M hydrochloric acid pH 2 solution, pH 3 citrate buffer or pH 4.5 sodium acetate buffer. The suspensions were equilibrated at 22 °C for 24 h under continuous stirring using appropriate magnetic stirrers to ensure a constant suspension temperature throughout the equilibration time. Three aliquots were taken from the equilibrated suspension using an electronic pipette, where one aliquot was used to confirm the temperature and the pH of the related suspension. The other two aliquots were transferred into thick-wall polycarbonate centrifuge tubes and inserted into an ultracentrifuge, Beckmann Coulter Optima™ MAX-XP, for analysis in duplicate. The ultracentrifuge was operated for the first 5 min until a temperature of 22 °C and the required vacuum were reached. Subsequently, the sample was centrifuged at 541’000-fold gravity for 10 min at a constant temperature of 22 °C. An aliquot of the supernatant was collected from the centrifuge tube using an electronic pipette after completion of the ultracentrifugation. The absence of particulate matter in this aliquot was confirmed for the finest nanocrystals by photon correlation spectroscopy (PCS) during method development. The aliquot was diluted with a fixed ratio of an organic solvent to robustly rule out any potential precipitation of the solubilized drug. Finally, this aliquot was analyzed for assay by high pressure liquid chromatography (HPLC), Agilent 1290 Infinity LC using a validated method with an analytical precision of better than 0.3 % relative standard deviation and a simultaneously operated system suitability test. In addition, the stability of the solubility of nanocrystals was investigated by applying extended equilibration times using the sample preparation and analytical method as described above.

X-ray powder diffraction (XRPD) was performed using a Rigaku, Smart Lab II in either reflection or transmission mode. For both modes, an angular range between 2° to 40° 2*θ* was scanned with an angular step of 0.017°. Cu-Kα radiation at 40 kV and 40 mA was used throughout the measurements. In reflection mode, 10.00 ± 0.05 mg of powder was prepared on a low background Si-sample holder for each sample. The sample holders were spun at 2 Hz during the measurement. A scan speed of 1.7 °/min was used. Samples were measured in triplicate to obtain an average signal, whereby for each measurement, a new sample holder was prepared. In transmission mode, a 0.7 mm OD glass capillary with 80 to 100 mm length was used. Nanocrystal suspensions were filled into the capillaries using a special 0.5 mm OD cannula. Filled cannulas were immediately afterwards closed with a small plug of malleable waxy material. Capillaries were spun with 2 Hz and a scan speed of 0.1 °/min was used.

### Modeling methods

The diffusion-limited dissolution of solids in a surrounding liquid phase is described by the Noyes-Whitney / Nernst-Brunner equation [62,63]:


(1)

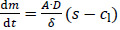



The dissolution rate d*m*/d*t* is a function of the surface area of the solids *A*, the diffusion coefficient of the solute *D*, the diffusion boundary layer thickness around the solid *δ*, the saturation solubility of the solute *s* and the time-dependent concentration in the liquid phase *c*_l_. This diffusion-based approach assumes saturation conditions at the solid’s surface followed by diffusion of the solute molecules through the diffusion boundary layer into the surrounding liquid with concentration *c*_l_, assuming a linear concentration gradient across the diffusion boundary layer.

The diffusion coefficient *D* is calculated according to the Stokes-Einstein equation:


(2)

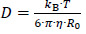



where *k*_B_ is the Boltzmann constant, *T* the temperature, *η* the dynamic viscosity of the liquid and *R*_0_ the hydrodynamic radius of the diffusing molecule. *R*_0_ is calculated based on the molar volume of the dissolving compound *V*_m_, assuming a spherical geometry of the diffusing molecule.

The diffusion boundary layer thickness is described by Prandtl’s boundary layer theory for macroscopic systems, in which the thickness of the diffusion boundary layer depends on the velocity of the surrounding liquid in relation to the solid surface [64]. This concept is no longer applicable for particles smaller than 1 μm since the particles practically follow the liquid flow due to a lack of inertia and the relative velocity approaches zero [65,66]. The exact behavior of the diffusion boundary layer thickness for particles smaller than 1 μm is not fully understood. However, the most frequent and accepted approach assumes a diffusion boundary layer thickness equal to the particle radius for such small particles [65,67-71]:


(3)

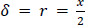



The increase of the solubility with the decrease in particle size is described by the Ostwald-Freundlich equation [72]:


(4)

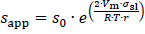



where *s*_app_ is the apparent solubility, *s*_0_ the thermodynamic solubility for coarse particles *x≫* 1 μm, *σ*_sl_ the interfacial energy between solid and liquid phase, *r* the particle radius, *V*_m_ the molar volume of the dissolving compound, *R* the universal gas constant and *T* the temperature. According to equation (4), the apparent solubility increases with decreasing particle size. This effect becomes notable for particles *x≪* 1 μm and has been experimentally demonstrated [8,43-46].

A numerical model was established, combining equations (1), (3) and (4) to describe the dissolution of a sample of small particles *x* ≪ 1 μm. Any true sample of particles is polydisperse and not monodisperse. Therefore, realistic samples should be described by a particle size distribution. Each particle within the particle size distribution with its specific particle size exhibits its own specific apparent solubility. Depending on the particle size distribution, the apparent solubility can vary notably from one end of the particle size distribution to the other. Therefore, the entire particle size distribution must be considered to accurately describe the dissolution of a particle system with *x* ≪ 1 μm.

A particle size distribution with appropriate discretization is required for the numerical model to minimize the variability of the calculated results. PCS measurements yield the mean particle size (*x*_PCS_) and the polydispersity index (PI). Based on the refractive index of the compound and the liquid, the volume-based (Q_3_) particle size distribution data can be obtained as percentiles for *x*_10,3_, *x*_20,3_ to *x*_80,3_, *x*_90,3_. Finally, these data are fitted using a log-normal function and discretized in 999 classes of the volume-based (Q_3_) particle size distribution, obtaining the percentiles *x*_0.1,3_, *x*_0.2,3_ to *x*_99.8,3_, *x*_99.9,3._. While the fitted log-normal distribution matches well the percentiles from *x*_10,3_ to *x*_90,3_, it should be noted that a non-negligible uncertainty is introduced specifically for the ranges below *x*_10,3_ and above *x*_90,3_ as here, an extrapolation of the experimental data is conducted as the true particle size distribution is not known.

The following assumptions are made for the numerical model:

- diffusion-controlled dissolution from a planar surface with linear concentration gradient across the diffusion boundary layer,
- perfectly mixed liquid phase with constant temperature,
- spherical particles, ideally dispersed in the dissolution medium,
- no drug-drug or drug-excipient interactions,
- no precipitation/crystallization / Ostwald ripening, no agglomeration.

**Figure 1.**
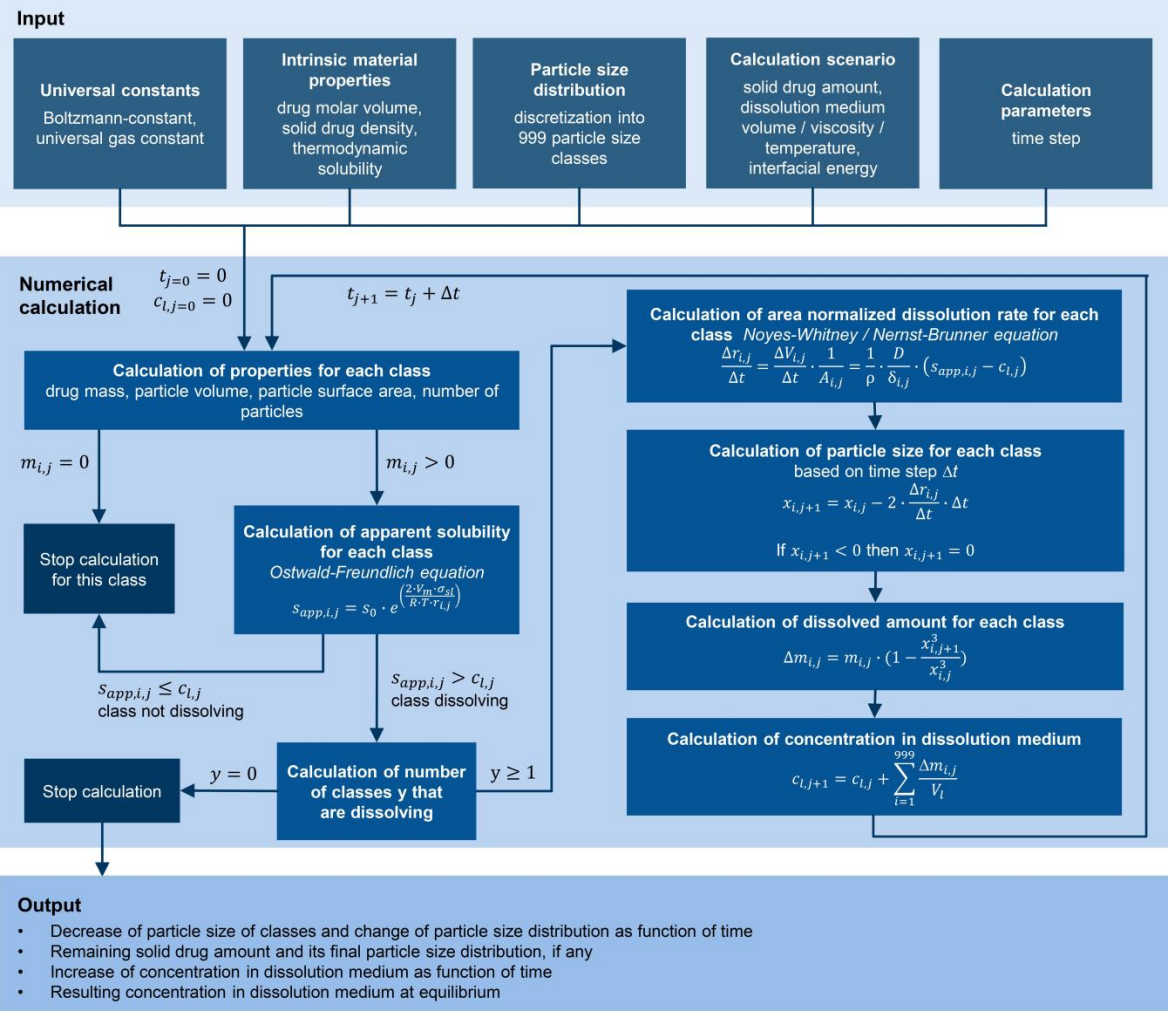
Scheme for the numerical calculation of nanocrystal dissolution considering the Ostwald-Freundlich and Noyes-Whitney / Nernst-Brunner equations with parameters: time *t*, time step ∆*t*, drug concentration *c*_l_, drug mass m, thermodynamic solubility for *x* ≫ 1μm, *s*_0_, apparent solubility *s*_app_, particle size *x*, particle radius *r*, drug molar volume *V*_m_, interfacial energy between solid drug and liquid phase *σ*_sl_, universal gas constant *R*, temperature *T*, particle volume *V*, particle surface area *A*, diffusion coefficient *D*, density of solid drug *ρ*, diffusion boundary layer thickness *δ*, volume of dissolution medium *V*_l_.

Figure 1 schematically outlines the workflow of the established numerical model for the calculation of the dissolution of small particles, specifically for size distributions with particles *x* ≪ 1 μm. Besides the input parameters needed for the equations (1), (2) and (4), as well as the particle size distribution data, information about the dissolution scenario, *i.e.*, the amount of solid drug and amount of dissolution media, is required as input parameters for the calculation. Thus the model offers the possibility to flexibly adjust the calculated scenario to any application of interest, *i.e.*, from sink to non-sink conditions. In this context, the Dose number (*D*o) is a functional parameter to simply describe the ratio of available solid drug to the thermodynamically soluble amount in a certain amount of dissolution media. *D*o was introduced by Amidon et al. in the 1990s [73,74] and the parameter has become a standard to assess and classify the risk for oral absorption in terms of dissolution [75-80].


(5)

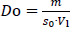



Where *m* is the amount of solid drug, *s*_0_ the thermodynamic solubility for large particles *x≫* 1 μm in the related dissolution medium with volume *V*_l_. The numerical calculation is carried out iteratively for each particle class *i*, with the number of iterations *j* and the time step *∆t*. As the first step in each iteration, the mass, volume, and surface area are calculated for each particle class. As long as the mass of a particle class is *m*_*i,j*_ > 0, the apparent solubility for each class *s*_*app,i,j*_ is calculated based on its particle size *x*_*i,j*_ using the Ostwald-Freundlich equation. If the apparent solubility is higher than the concentration in the dissolution medium *s*_app*,i,j*_ > *c*_*l,j*_, the particles of that class can further dissolve, and the dissolution rate is calculated using the Noyes-Whitney / Nernst-Brunner equation. With the selected time step *∆t*, the new particle size for each class for the next iteration *x*_*i,j+1*_ is determined as well as the amount of dissolved compound for each class *∆m*_*i,j*_. Consequently, the number of particles per class is constant and each particle remains in its initial class, however, the class’s particle size is decreasing. The sum of dissolved drug compound from all classes *∆m*_*j*_ is added to the concentration *c*_*l,j*_ resulting in the new concentration *c*_*l,j+1*_. Finally, the time for the next iteration step is determined *t*_*j+1*_ = *t*_*j*_*+∆t* . The calculation cycle is repeated as long as there is a particle class remaining for which *s*_*app,i,j*_ > *c*_*l,j*_ is true or until all material has been dissolved. At the end of the calculation, the system is considered to be in equilibrium. For simplification, throughout this manuscript, the term “thermodynamic solubility” is used to represent the solubility of coarse particles with a size x >> 1 μm, and the term “apparent solubility” is used interchangeably to “resulting concentration at equilibrium”, if supersaturation has been reached. Table 1 summarizes the input parameters used for the numerical model. The calculation was programmed in Microsoft Excel 2016 with Microsoft Visual Basic for Applications 7.1.

The decrease of the particle size of the individual classes and the change of the particle size distribution as a function of time is obtained as output from the numerical model. In addition, for cases where *D*o > 1, the remaining mass fraction of the solid drug and its final particle size distribution is captured, if applicable. Similarly, the concentration increase in the dissolution medium as a function of time as well as the equilibrium concentration in the dissolution medium, which corresponds to the achieved apparent solubility for cases where *D*o > 1, are obtained.

## Results and discussion

### Particle size characterization of nanocrystals

The coarse crystal and the manufactured nanocrystal particle size distributions as determined by laser light diffraction (LLD) and photon correlation spectroscopy (PCS) approximated into log-normal distributions are outlined in Table 2. The volume-based (*Q*_3_) percentiles *x*_10,3_, *x*_50,3_ (median) and *x*_90,3_ of the particle size distributions are reported, including the individual relative difference compared to the experimental results as obtained from LLD and PCS analysis. Further, the standard deviation (σ) of the approximated log-normal distribution is reported. The uncertainty for the ranges below *x*_10,3_ and above *x*_90,3_ must be highlighted as here experimental results were extrapolated. However, the obtained difference by the approximation of the log-normal distribution fit is considered appropriate. It should be noted that analytical centrifugation is presumed to be a more accurate technique to characterize the entire nanocrystal particle size distribution. The obtained results of the nanocrystal suspensions show the wide range of particle size distributions manufactured, well within the range of conventional products above 150 nm and within the sub-100 nm range. The finest nanocrystal particle size distribution was obtained for experiment No. 8, with most particles below 100 nm. Interestingly, the standard deviation for the wet media milled suspensions, including experiments No. 2 to 8 is quite comparable.

**Figure 2.**
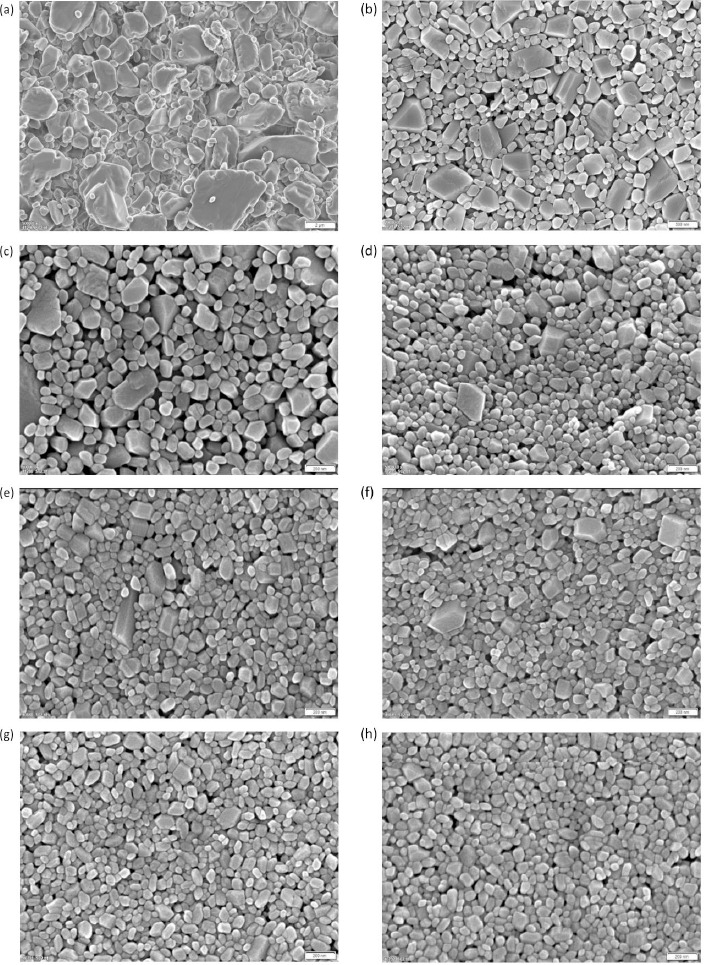
Micrographs of nanocrystals as determined by Scanning Electron Microscopy (SEM) corresponding to Table 2. **(a)** Experiment No. 1, x_50,3_ = 2443 nm. **(b)** Experiment No. 2, *x*_50,3_ = 261 nm. **(c)** Experiment No. 3, *x*_50,3_ = 201 nm. **(d)** Experiment No. 4, *x*_50,3_ = 140 nm. **(e)** Experiment No. 5, *x*_50,3_ = 111 nm. **(f)** Experiment No. 6, x_50,3_ = 93 nm. **(g)** Experiment No. 7, *x*_50,3_ = 90 nm. **(h)** Experiment No. 8, *x*_50,3_ = 79 nm; note the different levels of magnification of the micrographs.

The corresponding scanning electron micrographs of the manufactured nanocrystals are shown in Figure 2. The different levels of magnification of the micrographs should be noted. The coarse crystals are shown in Figure 2(a) and correspond to experiment No. 1. Coarse crystals with a particle size of about 5 μm and fine crystals below 1 μm can be seen in the micrograph and match well to the quantitative particle size distribution according to Table 2. Nanocrystals manufactured by wet media milling are shown in Figure 2(b) to (h). Even at the highest magnification, nanocrystals demonstrate throughout all experiments quite smooth surfaces. This observation corresponds to other investigations where the surface of wet media milled organic crystals is also determined by the solid-liquid equilibrium and not merely by ordinary mechanical fracture [81]. Figure 2(b) shows the coarsest nanocrystals manufactured by wet media milling with one larger sized population of irregular, square-shaped particles with smooth planes and a second smaller-sized population of irregular, round-shaped particles. Interestingly, the population of irregular, square-shaped particles is decreasing with further size reduction of the nanocrystals, as can be seen upon the evolution from Figure 2(c) to (h). The population of irregular, square-shaped particles with smooth planes no longer exists for the smallest nanocrystals obtained, see Figure 2(h). Most of the particles of the finest manufactured nanocrystals in Figure 2(h) are well below 100 nm and correspond well with the quantitative results in Table 2. The entire nanocrystal population shows irregular, rounded-shaped particles and even ultrafine nanocrystals with almost sphere-like shape. On closer examination, ultrafine nanocrystals with a particle size of about 20 nm can be found.

### Solid-state characterization of nanocrystals

Nanocrystals can be seen as an intermediate step in the continuum between perfectly crystalline materials and amorphous phases, where defects and surface features of the crystalline structure can no longer be ignored when describing the solid state of the particles [82].

**Figure 3.**
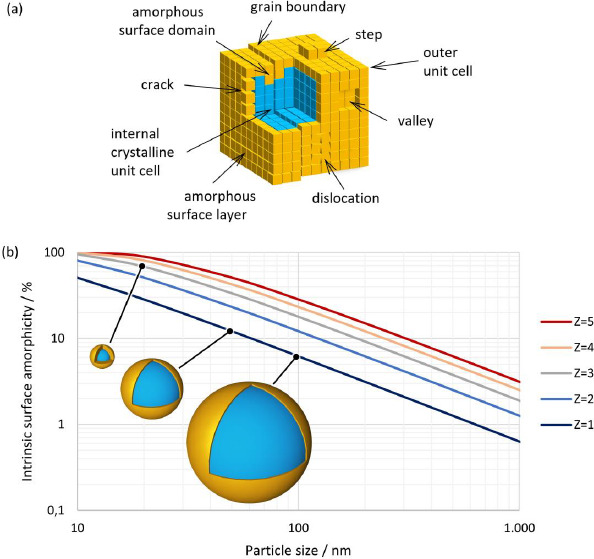
**(a)** Schematic representation of a hypothetical crystal with defects. **(b)** Intrinsic surface amorphicity (AM) of a spherical particle with particle size (*x*) and depth of amorphous surface layer in a number of layers of unit cells (*Z*).

In general, crystalline particles might appear smooth by light or electron microscopy. On a molecular scale, however, every particle will show a certain amount of surface rugosity due to the growth patterns of the crystal. This might lead to steps or valleys on the surface of a particle. In addition, during crystallization, imperfections such as surface cracks, grain boundaries between crystallites and dislocations can also create a larger actual surface area, see Figure 3(a). It should be noted that some of these contributions might not be accessible, with every instrumental technique giving rise to different estimates depending on the chosen methodology. Further, noise from sample containers and inactive ingredients in the sample will generate a background signal that will limit the sensitivity of any method chosen.

Crystalline substances show a distinct XRPD pattern. However, by definition, at least the outermost molecular layer on the surface of a crystal, see Figure 3(a) orange color, cannot exhibit the crystal lattice that defines the bulk crystal, as both near and far orders are absent at the surface. It can be assumed that this layer might be fully amorphous. Similarly, the second, and maybe the third, outermost layers do not have the same amount of far order as unit cells deep inside the crystal, see Figure 3(a) blue color.

Fine particles in the lower micron-sized or even submicron-sized range will exhibit substantially more relative surface area that would thereby naturally increase the amorphous fraction of the material, even in the absence of further defects. Estimating this fraction would therefore predict a likely minimum amount of amorphous content that could be expected depending on the particle size if the surface layer were truly fully amorphous. The amount of amorphous surface layer (*AM)* in relation to the particle volume can be calculated using a simplified core-shell model of concentric spheres with a shell thickness corresponding to the number of layers of unit cells according to the expression below.


(6)

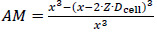



whereby *D*_cell_ is the diameter of the crystallographic unit cell, *x* the particle size, and *Z* is the depth of the amorphous surface layer expressed in layers of unit cells. Using the volume of the unit cell *V*_cell_, which can be calculated using the generalized expression:


(7)

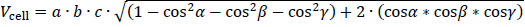



Whereby *a*, *b* and *c* are the lengths of the unit cell axes and *α*, *β* and *γ* are the respective angles. The small average particle size and the significantly rounded edges of individual particles justify the approximation of spherical particle morphology. Table 3 shows the estimated percentage of the intrinsic amorphous surface for several relevant particle sizes and layer depths. It can be seen that for coarse particles, assumed to have a particle size of 100 μm, the intrinsic amorphous surface layer percentage is negligible, even at a depth of three layers. For particle sizes between 1 and 3 μm, the intrinsic surface amorphicity is estimated to be not more than 2 %, likely below the limit of detection with state-of-the-art equipment.

For particles with a size below about 200 nm, the intrinsic amorphous surface contribution is estimated to become detectable and quantifiable with state-of-the-art equipment. On average, between 6.2 to 17.8 % of amorphous content due to the surface layers are estimated for a 100 nm particle. Ultrafine particles of 20 nm are likely to be more amorphous than crystalline. The relationship between particle size and the intrinsic amorphous surface is depicted in Figure 3(b). Further, the inserts visualize the amorphous layer for a few selected particle sizes and layer depths.

Transmission XRPD experiments using a glass capillary were tried to quantify the amorphous content of the nanocrystals. The glass capillary and the placebo create a background signal that needs to be subtracted. It was assumed that all glass capillaries were identical and that the placebo solution of the stabilizers could be subtracted from the total signal of the suspension. However, prior investigations had shown that part or all of the stabilizer interacts with the surface of the wet-media milled particles and is therefore bound and no longer freely available in the supernatant solution. This bound amount should vary with the particle size, but it was assumed that the variation between the individual batches was small. To further investigate this effect, a suspension with the smallest mean particle size was centrifuged using an ultracentrifuge generating a particle-free supernatant solution. This supernatant showed a low XRPD absorption across the angular range, showing that most of the stabilizer is indeed absorbed to the surface of the particles in suspension (data not shown). The centrifuged placebo solution was used for the background correction for all suspensions.

**Figure 4.**
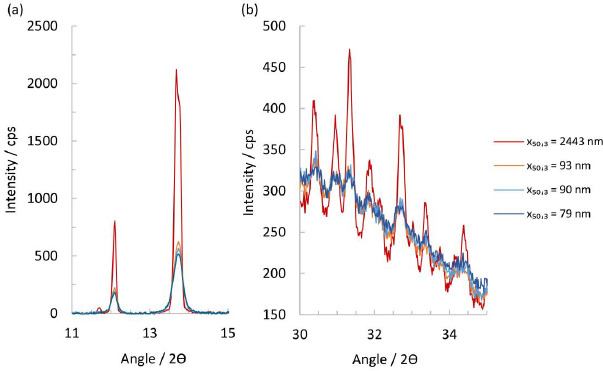
Transmission XRPD patterns at selected diffraction angles for experiment No.1 and 6 to 8 with particle size *x*_50,3_ of 2443, 93, 90 and 79 nm. **(a)** Transmission XRPD pattern between 11° and 15° 2 *ϴ*. **(b)** Transmission XRPD pattern between 30° and 35° 2*ϴ*.

Scrutinizing the XRPD patterns in the range of 11 to 15° 2ϴ shows that the suspension containing the micron-sized particles has a significantly more intense reflection signal than the nanocrystal particles, see Figure 4(a). Indeed, a small diffraction peak at 11.7° is still visible that usually can only be seen in a fully crystalline material. For the nanocrystal particles of *x*_50,3_ of 79, 90 and 93 nm, the intensity of the peak in this range diminishes with their respective particle size, and a visible peak broadening compared to the micron-sized particles can be seen. At higher 2*ϴ* angles, the micron-sized particles again show the highest residual peak intensity and a slightly smaller halo intensity, while the other nanocrystal particles appear to be identical. At high diffraction angles, *i.e.*, 30° to 35° 2*ϴ*, some patterns show a residual amorphous halo after subtraction of the placebo, indicating a significant degree of amorphous content in the nanocrystals.

For the quantification of the amorphous content, a fully crystalline and a fully amorphous reference in the same medium are necessary. Unfortunately, suspension of unmilled material or amorphous drug substance could not be generated. The unmilled, nominally 100 % crystalline material does not suspend in the aqueous medium, while the amorphous material showed rapid Ostwald ripening and recrystallization. Consequently, no estimate for the amorphous content of the suspensions can be given. However, using the Scherrer equation, one can calculate an average crystallite size using a crystalline silicon standard (NIST® SRM® 675) as a reference. Table 4 shows the peak parameters obtained from the pattern, the calculated crystallite size and the known median particle size *x*_50,3_. The average peak height is steadily diminishing with a smaller median particle size. The nanocrystals of experiments No. 6 to 8 have a very similar, if not identical, average crystallite size of approximately 31 nm.

Plotting the peak height at 13.7° against the measured particle size in a semi-logarithmic plot allows fitting a linear trendline through the data, whose root predicts a likely particle size at which the peaks’ diffraction signal would be merging with the amorphous background. The extrapolation estimates that below a particle size of approximately 21 nm, the drug substance would become X-ray amorphous, with the exact nature of the particles, *i.e.*, crystalline or amorphous, being undeterminable. It should be noted that a crystal of 21 nm would still contain about 7000 unit cells. However, about 28 %, *i.e.*, 2000 of those would already constitute a single surface layer and three layers would amount to 68 %, notwithstanding internal dislocations and grain boundaries that would further reduce any diffraction signal. Therefore, a more appropriate limit of detection of crystallinity by transmission XRPD should be above a particle size of 21 nm.

It should be kept in mind that the samples actually have a particle size distribution and that the XRPD signal is a superposition of the individual contributions of each particle size in the sample. Indeed, an SEM of a sample with a median particle size *x*_50,3_ = 79 nm shows that the sample exhibits particles that represent particle sizes according to the determined particle size distribution, see experiment No. 8 in Table 2 and SEM in Figure 2(h). However, nanocrystal particles in the vicinity of 30 nm and even around 20 nm can be found in the high magnification SEM in Figure 2(h).

**Figure 5.**
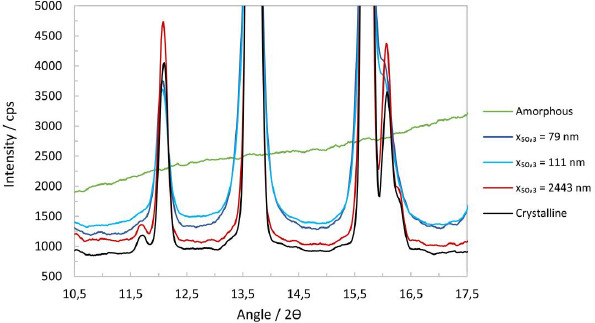
Reflection XRPD patterns between 10.5° to 17.5° 2θ, with 100 % crystalline drug substance, 100 % amorphous drug substance and for experiments No.1, 5 and 8 with particle size *x*_50,3_ of 2443 nm, 111 nm and 79 nm.

The liquid nature of the suspensions makes handling and measurement awkward for XRPD investigations in reflection mode. It is preferable to handle the samples as dry powders. To obtain dry powder, one milliliter of selected suspensions was dried at 50 °C in an oven for a week. The solid residue obtained after drying was ground into a fine powder in an agate mortar. In addition, the reflection measurements allow the generation of a calibration line with respect to the amorphous content by using unmilled crystalline material and amorphous drug substance as 100 % and 0 % crystalline reference, respectively. Further, physical mixtures of these allow the preparation of calibration mixtures with known amorphous content. The suspensions also contain 4 %w of stabilizer, which relates to 13.79 %w in the dried samples. Therefore, the patterns obtained were corrected for their stabilizer contribution. Each sample was measured in triplicate and an average of the three individual datasets was used to minimize intensity errors. Further, each sample was weighed to 10.0 ± 0.050 mg to minimize intensity variations due to differences in mass absorption between samples. The stabilizer contribution was subtracted from the suspension raw data, and the pure solid samples, *i.e.*, unmilled crystalline, amorphous and micron-sized samples, were weighted accordingly. The pattern of the Si-holder was subtracted subsequently, and the resulting patterns were smoothed with a 9-point straight line Savitzky-Golay window, resulting in a less noisy pattern overall, see Figure 5.

**Figure 6.**
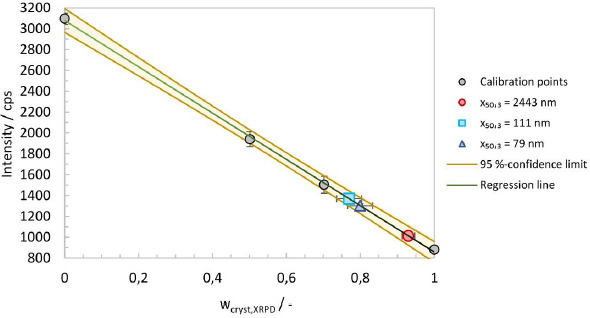
Calibration line at 16.9994° 2θ by reflection XRPD for the calculation of the amorphous content (w_cryst,XRPD_) with 95 %-confidence limits and results for calibration points and experiments No.1, 5 and 8 with particle size *x*_50,3_ of 2443 nm, 111 nm and 79 nm.

The physical mixtures together with the crystalline and amorphous material, allowed the generation of a calibration line by plotting the crystalline content against the baseline intensity. The area around 17° 2θ is considered suitable for analysis. A calibration line at 16.994° 2θ was generated, including regression and error analysis. The intensity error was calculated as the standard deviation of three measurements for the four calibration mixtures, see Figure 6. The regression line and the 95 %-confidence limits were calculated using Microsoft Excel 2016. An excellent fit and high regression coefficient give credence to the assumption that the baseline intensity is indeed linearly correlated to the crystalline mass fraction.

The crystalline content (*w*_cryst,XRPD_) for experiments No. 1, 5 and 8, as well as the errors, were calculated from the regression parameters and are summarized in Table 5. It should be noted that the amorphous content (*w*_amorph,XRPD_) is defined according to the following equation.


(8)

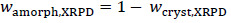



The intrinsic amorphous surface layer contribution for a depth of one unit cell was calculated using the median particle size (*w*_amorph,Z=1_), see Table 5. Results for the micron-sized drug substance and the nanocrystal drug substance batches cluster separately, suggesting that the nanocrystals do have a higher amorphous content than the micron-sized drug substance. Within the error limits, the micron-sized drug substance shows an amorphous content of 8 %w, while the nanocrystal drug substance batches show an amorphous content of 21.5 %w on average. Both values are significantly larger than those suggested by assuming a single amorphous surface layer of unit cells, see Table 5.

The contribution from the nanocrystals to the estimated intrinsic surface amorphicity can be calculated by taking the whole particle size distribution from Table 2 into account. This should give a more accurate estimate of the intrinsic surface amorphicity. Assuming several layers of non-crystalline surface unit cells (Z), the intrinsic surface amorphicity (*w*_amorph,Z_) contribution rises significantly depending on the number of layers of unit cells. It should also be kept in mind that the limit of detection of crystallinity, determined by reflection XRPD, suggests that particles in that size range might still be crystalline, although appearing amorphous in the measurement. Therefore, the true amount of amorphicity could be less than the measured one. The nanocrystal particle size distribution data suggests that no more than 1 %w of the particles would fall into the size range below 20 to 30 nm for the smallest average particle size, *i.e.*, experiment No. 8.

The difference between the estimated intrinsic surface amorphicity for a single amorphous layer and the calculated amorphicity based on XRPD measurements is only about a factor of 2 to 4 for nanocrystals and is therefore reasonable, see experiment No. 5 and 8 in Table 5 and Table 6. However, it is more prudent to assume that the high curvature of the nanocrystal particles might impact the crystal structure deeper than just the mere surface and at least 1 or 2 additional layers are affected. Indeed with *Z*=3, there is a good match between calculated and measured amorphous content. It should be kept in mind that a depth of three unit cells would only correspond to about 3.3 nm.

On the other hand, the large discrepancy between XRPD measurements and the estimation for the micron-sized coarse crystals by a factor of 27 is striking, see experiment No. 1 in Table 5. The material was processed by dry jet milling. This process is operated in a gaseous atmosphere and breakage is obtained by elastic and elastic-plastic deformation. Therefore, damaged material may be able to recrystallize by its intrinsic properties and elevated surface temperature from the moving crack. In contrast, nanocrystals were manufactured by wet-media milling, which is operated in a liquid medium and breakage can be assumed to take place for submicron-sized particles by plastic deformation. Damaged material may be able to recrystallize by its intrinsic properties but also by solubilization and precipitation at the solid-liquid interface [81]. Unfortunately, the interplay of the different phenomena and resulting product properties is not understood for dry-jet milling and wet-media milling. However, considering the theoretical model outlined earlier, the obtained results for dry-jet milled particles suggest an amorphous surface layer of about 32 surface layers of unit cells or a depth of 35 nm. Alternatively, it can be hypothesized that the X-rays penetrate the crystal as a whole and the resulting pattern might also contain contributions from dislocations and grain boundaries within the crystals, thereby possibly giving rise to an elevated estimate of the amorphous content without an increased intrinsic surface amorphicity.

### Experimental results for apparent solubility

The apparent solubility was experimentally determined (*s*_app,exp_) for all polydisperse nanocrystal suspensions listed in Table 2 in pH 3 citrate buffer for *D*o = 8.9. In addition, the apparent solubility was experimentally determined for a wide range of *D*o values from 2.7 to 179 for the nanocrystal suspension with *x*_50,3_ = 111 nm in pH 3 citrate buffer. Table 7 summarizes the results of both sets of experiments. The relative difference for the duplicate analysis of all samples was identified with a maximum of 4 %, highlighting the satisfactory analytical precision of the established apparent solubility values, particularly taking into consideration the challenging nanocrystal separation step.

For different particle size distributions with a constant *D*o, a systematic increase of the apparent solubility with decreasing median nanocrystal particle size *x*_50,3_ is observed, as described by the Ostwald-Freundlich equation. The apparent solubility increases from the initial coarse crystal suspension to the finest nanocrystal particle size distribution by almost a factor of 2. As a general point, the determined increase of the apparent solubility compared to the thermodynamic solubility is a matter of true supersaturation for this specific drug-stabilizer system. The increase in apparent solubility by almost a factor of 2 is significantly higher compared to so far reported values [8,43-46]. This is reasonable due to the significantly finer drug particle size distributions evaluated in this study. It can be noted that the increase in apparent solubility with decreasing particle size becomes significant for the studied drug compound only for *x*_50,3_ << 1 μm. The characteristic particle size where the increase of apparent solubility due to the decrease of particle size becomes significant depends on the specific drug-stabilizer system. This includes the finest particle size and the related amount of fines of the drug particle size distribution, the interfacial energy between solid and liquid σ_sl_, the drug molar volume *V*_m_, and the stability of the supersaturation regarding precipitation and Ostwald ripening. The definition of a general particle size threshold, below which solubility increases due to decreasing particle size becomes significant, is therefore not possible. Instead, this question must be evaluated for each specific drug-stabilizer system.

Interestingly, a dependence of the apparent solubility on *D*o is identified for the same nanocrystal particle size distribution, see experiments No. 9 to 15 in Table 7. While the apparent solubility increases notably with increasing *D*o in the lower *D*o range up to a value of *D*o = 18, a plateauing is observed for the higher Do range. The Ostwald-Freundlich equation considering *x*_50,3_ alone is not sufficient for the explanation of this behavior, as the entire particle size distribution and its evolution during dissolution need to be considered. While for systems with *x* ≫ 1 μm, an increase in *D*o only results in an increase of available liquid-solid surface area for dissolution and thereby faster dissolution kinetics, the implications for systems with *x* ≪ 1 μm are more complex, finally impacting not only the available liquid-solid surface area but also the shape of the particle size distribution at the end of the dissolution process and thereby the resulting apparent solubility. A simplified thought experiment might facilitate the understanding of the observed behavior. For two theoretical scenarios with *D*o values of 3 and 20, the dissolved solids at equilibrium will be roughly 1/3 and 1/20, respectively. Assuming that the small particles dissolve first due to their size and increased apparent solubility, the smallest remaining particle after dissolution would correspond to *x*_33,3_ and *x*_5,3_, respectively, neglecting, for now, the size reduction of those particles during the dissolution process. Since *x*_5,3_ < x_33,3_, the measured apparent solubility for the scenario with *D*o = 20 is expected to be higher compared to *D*o = 3. Other studies have also varied Do in different scenarios [31,54]. However, the complex implications of this parameter were not always fully considered.

### Numerical model

The interfacial energy between solid and liquid remains an unknown parameter since there are no analytical techniques available to directly characterize the interfacial energy between drug nanocrystals in suspension that contains stabilizers absorbed on its surface and the aqueous liquid media containing solubilized stabilizers. The neat drug substance was characterized utilizing the drop shape analysis method in an attempt to identify a plausible range for the interfacial energy by which a surface free energy of 65.5 ± 5.5 mJ/m^2^ was determined, comprised of a disperse part of 49.5 ± 1.1 mJ/m^2^ and a polar part of 16.0 ± 4.5 mJ/m^2^. The complementary analysis by Inverse Gas Chromatography resulted in a disperse part of the interfacial energy of 55 mJ/m^2^. It can therefore be concluded that the interfacial energy σ_sl_ between drug nanocrystals and the liquid dissolution media can be estimated to be considerably below 65 mJ/m^2^, due to the action of interfacial energy reducing stabilizers.

**Figure 7.**
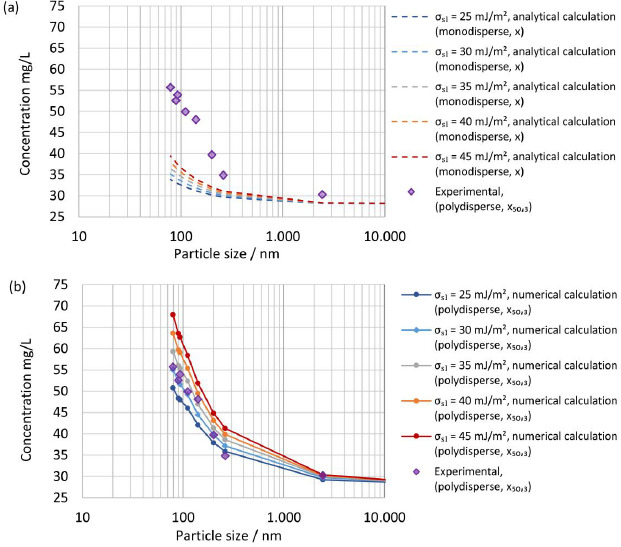
Experimentally determined apparent solubility (*s*_app,exp_) of polydisperse particle size distributions according to Table 7 in pH 3 dissolution media at constant ratio of drug/dissolution media (*D*o = 8.9) vs theoretical calculated apparent solubility (*s*_app,th_) for selected interfacial energies plotted against particle size. (**a)** Comparison of theoretical apparent solubility (*s*_app,th_) with monodisperse particle size *x* = *x*_50,3_ using analytical calculation (equation (4)) vs experimentally determined apparent solubility (*s*_app,exp_) with polydisperse particle size distribution. **(b)** Comparison of theoretical apparent solubility (*s*_app,th_) with polydisperse particle size distributions according to Table 2 using numerical calculation vs. experimentally determined apparent solubility (*s*_app,exp_) with polydisperse particle size distribution.

Systematic calculations were carried out with selected interfacial energies in the range below 65 mJ/m^2^ to select interfacial energy for the numerical calculation. A comparison of the experimentally determined apparent solubility from polydisperse particle size distribution, according to experiments No. 1 to 8 in Table 7, with the theoretical apparent solubility calculated analytically using equation (4) assuming a monodisperse particle size for selected interfacial energies is displayed in Figure 7(a). The median particle size *x*_50,3_ was selected as the monodisperse particle size *x* for the calculations. The calculated theoretical apparent solubility significantly underpredicts the experimentally determined apparent solubility for all selected interfacial energies demonstrating the clear limitations of assuming a monodisperse particle size and neglecting the change of particle size distribution during dissolution for a drug-stabilizer system containing drug particles with a particle size well below 1 μm.

Figure 7(b) shows a comparison of the experimentally determined apparent solubility from polydisperse particle size distribution, according to experiments No. 1 to 8 in Table 7, with the theoretical apparent solubility calculated using the established numerical model and selected interfacial energies. The experimentally determined and calculated apparent solubility is plotted against the median particle size x_50,3_. The consideration of the entire particle size distribution and its change during the dissolution process until the apparent solubility reaches equilibrium allows an appropriate description of the experimental data. The best agreement between experimental and calculated theoretical apparent solubility is found for interfacial energy of 30 mJ/m^2^, which was selected for all further calculations. Interestingly, the determined interfacial energy is in good agreement with the selected interfacial energy by Jinno et al. [83].

**Figure 8.**
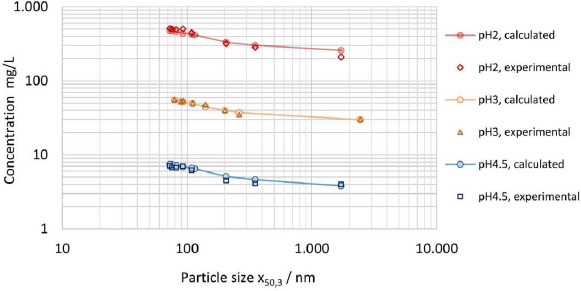
Experimentally determined apparent solubility (*s*_app,exp_) of polydisperse particle size distributions in pH 2, pH 3 and pH 4.5 dissolution media vs theoretical calculated apparent solubility (*s*_app,th_) for polydisperse particle size distributions according to Table 2 and interfacial energy of 30 mJ/m^2^ using numerical calculation with representation against median particle size *x*_50,3_.

In addition to the results reported in Table 7 for the apparent solubility determined in pH 3 citrate buffer, the apparent solubility was experimentally determined for 0.01 M hydrochloric acid pH 2 solution and pH 4.5 sodium acetate buffer, using partly the same but also additional nanocrystal suspensions (data not shown), while maintaining comparable *D*o values. Figure 8 displays the experimentally determined apparent solubility for all three dissolution media in comparison to the theoretical apparent solubility calculated by the numerical model using interfacial energy of 30 mJ/m^2^. The data set demonstrates the applicability of the model to various aqueous dissolution media with different levels of thermodynamic solubility across about two orders of magnitude. The relative change in solubility *s*/*s*_0_ as described by equation (4) is the same for any media, independent of the absolute value of the thermodynamic solubility if *D*o is kept constant. Consequently, the relative change of apparent solubility shown in Figure 8 is the same for all three investigated dissolution media. It should be particularly highlighted that the experimental and calculated results are quite conclusive according to the Noyes-Whitney/Nernst-Brunner and Ostwald-Freundlich equations. Interestingly, the results obtained for apparent solubility against drug particle size, see Figure 7, coincide in trend with the results obtained on the hypothesized intrinsic amorphous surface layer, see Figure 3(b) and Table 3. A potential relationship between the increase in apparent solubility and the increase of the hypothesized intrinsic surface amorphicity was not further investigated in this study.

**Figure 9.**
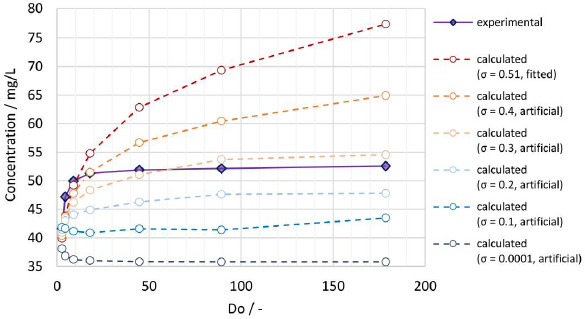
Experimentally determined apparent solubility (*s*_app,exp_) for a polydisperse particle size distribution (particle size *x*_50,3_ = 111 nm) with different ratio of available solids to thermodynamically soluble amount (*D*o) vs calculated apparent solubility (*s*_app,th_) for polydisperse particle size distribution with median particle size *x*_50,3_ = 111 nm with a fitted standard deviation of the particle size distribution (*σ*) of 0.51 and selected standard deviations of 0.4, 0.3, 0.2, 0.1 and 0.0001 with interfacial energy 30 mJ/m^2^ and different ratio of available solids to thermodynamically soluble amount (*D*o) using numerical calculation.

A comparison of the experimentally determined apparent solubility from the same polydisperse particle size distribution (*x*_50,3_ = 111 nm), applying different Do values according to experiments No. 9 to 14 in Table 7, with the theoretical apparent solubility calculated by the established numerical model is shown in Figure 9. Using the fitted width of the PSD *σ* = 0.51, the calculated apparent solubility increases with increasing Do values comparable to the observations for the experimental data, demonstrating that the numerical model can successfully capture this behavior, which would not be possible by assuming a monodisperse particle size. However, while the experimental data form a distinct plateau, the calculated apparent solubility keeps increasing and levels off much slower. For the calculation the interfacial energy value of 30 mJ/m2 was used, which was obtained by fitting data from experiments No. 1 to 8 with a fixed *D*o = 8.9. For lower *D*o values, the numerical model seems to underpredict the apparent solubility while overpredicting for higher *D*o values. Potential root causes for the observed discrepancy include: 1) no consideration of particle morphology (assumption of spherical particles), 2) uncertainty on experimentally determined and fitted particle size distribution, especially regarding the overestimation of the fine fraction.

Of these two potential root causes, the latter was investigated by systematic variation of the particle size distribution at constant median particle size *x*_50,3_. The standard deviation of the log-normal distribution function (*σ*) was varied from the value of 0.51, determined by fitting the log-normal distribution to the experimental data, to selected values of 0.4, 0.3, 0.2, 0.1 and 0.0001, artificially mimicking narrower particle size distributions and eventually a monodisperse system. The obtained calculated results are also plotted in Figure 9.

The lower the standard deviation, the lower the amount of the fine fraction, which is relevant for achieving supersaturation. Hence, the calculated apparent solubility decreases for decreasing standard deviations. A more pronounced plateau can be seen when the standard deviation reaches the artificial value of 0.3, which is in better agreement with the experimental data. For the monodisperse system (*σ* = 0.0001), the apparent solubility first decreases with increasing dose number before stabilizing at a constant value. For low dose numbers, the monodisperse particle size notably decreases during the dissolution process, leading to an increase in the apparent solubility. With increasing dose number, the decrease of the particle size and respective increase of the apparent solubility becomes smaller. Most importantly, these observations emphasize the high relevance of the particle size distribution as an input parameter for the calculation of the apparent solubility. An exact characterization of the drug particle size distribution, especially its fine fraction, is therefore paramount to model its dissolution behavior more accurately.

For this investigation, the experimental data from PCS measurements had to be further processed by fitting them to a log-normal distribution function and extrapolation below *x*_10,3_ introducing significant uncertainty. In fact, it can be seen in Table 2 that already the *x*_10,3_ value is systematically underpredicted by the fitted log-normal distribution function. This leads to an overestimation of the fine fraction in the numerical calculation, which leads to an overestimation of the calculated apparent solubility, especially for high Do values. Furthermore, the assumption of a log-normal shaped distribution function might not be ideal for the nanocrystal systems and fails to accurately describe the lower end of the true particle size distribution.

The accuracy of the numerical model and its capability to describe the dependence of the apparent solubility on Do could be further increased by improving the quality of the input data. Analytical centrifugation coupled with appropriate interpolation for sufficient discretization might be a better-suited method to describe the particle size distributions of nanocrystal systems.

Nevertheless, for this study, the fitted particle size distributions, as reported in Table 2, are used for further calculations without any artificial adjustment. A reasonable error of < 10 % between experimental and calculated apparent solubility is found for *D*o < 18, which is considered appropriate for further calculations. For most calculations carried out in this study, *D*o < 18 is fulfilled.

Since a true supersaturation is obtained during the dissolution of ultrafine drug nanocrystal particle size distributions for a solid drug amount at *D*o >1, subsequent precipitation/crystallization and decrease of the apparent solubility over time to the level of the thermodynamic solubility by way of Ostwald ripening would be a matter of concern. For this drug-stabilizer system, however, stability studies with selected drug nanocrystal suspensions have shown good stability on the level of supersaturation at 22 °C over several weeks with a decrease of the measured apparent solubility of less than 10 %.

### Modeling results for in vivo dissolution

Human gastrointestinal physiology is a complex interplay of different factors. Recent advances highlighted the dynamics within the gastrointestinal tract and the relevance of the parameters fluid volume and pH, and their related variabilities [84-86]. Modeling approaches are valuable and efficient to experiment *in silico* the complexity of different scenarios of formulation-gastrointestinal parameter combinations, provided the validity of the model. Different formulations and *in vivo* scenarios were selected to calculate the dissolution behavior using the established numerical model, representing a simplistic, closed system neglecting any further *in vivo* relevant process, *i.e.*, the evolution of fluid parameters and permeation of the drug. A fluid of pH 2 and a minimum volume of 20 mL and a maximum volume of 250 mL were selected to represent the variability of the fasted state of the gastrointestinal tract [84-86]. A fluid of pH 3 and 4.5 and a minimum volume of 250 mL and a maximum volume of 900 mL were selected to represent the variability of the fed-state of the gastrointestinal tract [84-86]. The formulation parameters solid drug amount, respectively dose strength and nanocrystal particle size distribution were investigated, which represent typical configurable variables in drug development. The solid drug amount, respectively dose strength, was selected to 25 and 100 mg. Different drug nanocrystal particle size distributions were selected according to experiments No. 1, 2, 5 and 8 in Table 2.

**Figure 10.**
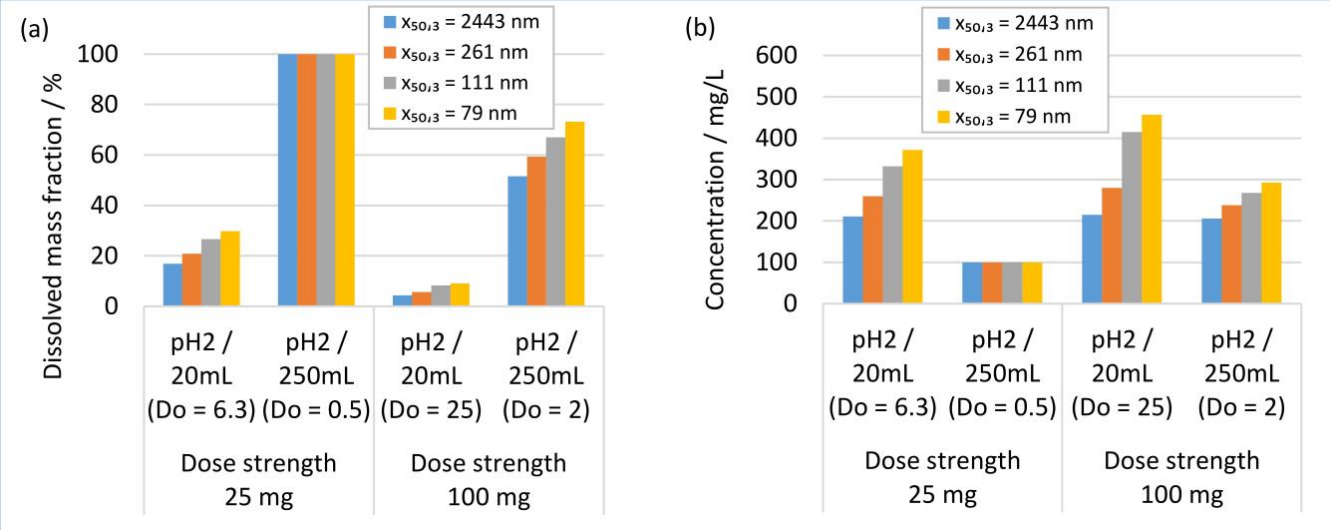
Calculated results for *in vivo* fasted-state scenarios of drug nanocrystal particle size distributions with *x*_50,3_ of 2443 nm, 261 nm, 111 nm and 79 nm according to Table 2, dose strengths of 25 mg and 100 mg in 20 mL and 250 mL fluid of pH 2. **(a)** Calculated results for dissolved mass fraction. **(b)** Calculated results for concentration at equilibrium.

**Figure 11.**
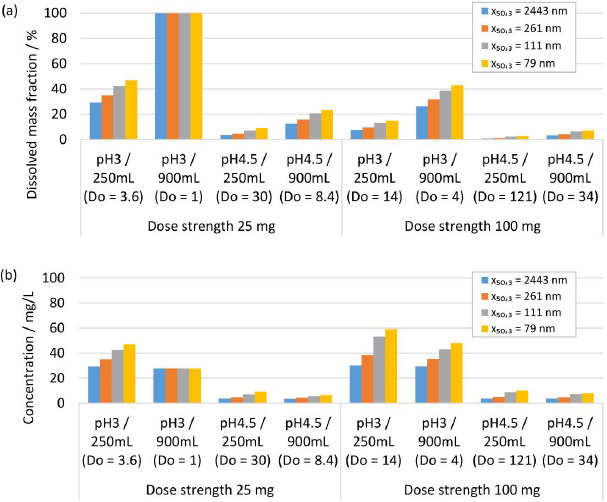
Calculated results for *in vivo* fed-state scenarios of drug nanocrystal particle size distributions with *x*_50,3_ of 2443 nm, 261 nm, 111 nm and 79 nm according to Table 2, dose strengths of 25 mg and 100 mg in 250 mL and 900 mL fluid of pH 3 and pH 4.5. **(a)** Calculated results for dissolved mass fraction. **(b)** Calculated results for concentration at equilibrium.

Figure 10 shows the *in silico* dissolution results obtained for the selected fasted-state parameter combinations. Figure 10(a) shows the dissolved mass fraction and Figure 10(b) the related concentration at equilibrium. The obtained *in silico* results for the selected fed-state parameter combinations are shown in Figure 11(a) for dissolved mass fraction and in Figure 11(b) for the related concentration at equilibrium. The drug nanocrystal particle size distributions are reported by median particle size *x*_50,3_. The dissolved mass fraction increases with decreasing *D*o (increasing fluid volume and decreasing dose strength) and with decreasing pH, due to the pH-dependent solubility (compare also Figure 8) of the investigated compound, see Figures 10(a) and 11(a). In addition, different nanocrystal particle size distributions show a notable impact on the dissolved mass fraction, yielding higher dissolved mass fractions with decreasing particle size. The values increase up to a factor of about 2 in accordance with the results reported above. Obviously, no impact from different nanocrystal particle size distributions is observed for scenarios with *D*o ≤ 1, where the thermodynamically soluble amount is equal or higher compared to the dose strength, see 25 mg dose strength in 250 mL fluid of pH 2, Figure 10(a) and 25 mg dose strength in 900 mL fluid volume of pH 3, see Figure 10(a). When looking at the concentration at equilibrium, see Figures 10(b) and 11(b), the effect of *D*o becomes visible (compare Figure 9). Although the dissolved mass fraction is lower for higher Do, the resulting concentration at equilibrium is generally increasing with *D*o. These *in silico* dissolution results are relevant for the *in vivo* performance since the concentration acts as a driving force for drug permeation. Consequently, the relative increase of solubility due to decreasing particle size and increasing *D*o may help elevate drug permeation and associated bioavailability.

The true supersaturation identified for this drug-stabilizer system at realistic *in vivo* conditions for ultrafine drug nanocrystal particle size distributions administered under conditions with *D*o >> 1 emphasizes the relevance for potential bioavailability improvement. The importance of supersaturation to promote drug permeation and *in vivo* absorption is extensively outlined in the context of amorphous solid dispersions as an alternative enabling formulation to improve the bioavailability of poorly-soluble drugs for oral administration [87-89]. Therefore, supersaturation by drug nanocrystals may contribute to bioavailability improvement in addition to the generally accepted factor of dissolution rate improvement by the increase of drug nanocrystal-specific surface area. Further mechanisms to promote potential bioavailability gains from ultrafine drug nanocrystals are adhesion to the gut wall and the penetration of drug particles of 20 to 100 nm [90]. However, the understanding of these further mechanisms is limited [90] and they are not subject to the present investigation.

### Modelling results for in vitro dissolution

**Figure 12.**
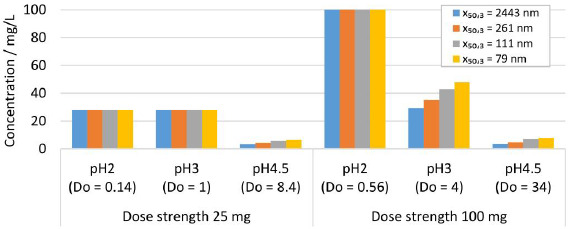
Calculated results for resulting concentration at equilibrium state for *in vitro* dissolution scenarios of drug nanocrystal particle size distributions with *x*_50,3_ of 2443 nm, 261 nm, 111 nm and 79 nm according to Table 2, dose strengths of 25 mg and 100 mg in 900 mL dissolution media of pH 2, pH 3 and pH 4.5.

Further *in silico* studies were conducted to investigate the dissolution behavior of drug nanocrystals during *in vitro* dissolution testing. A standard dissolution test set-up of 900 mL dissolution media with pH 2, 3 and 4.5 was considered. The formulation parameters solid drug amount, dose strength of 25 and 100 mg and various nanocrystal particle size distribution were selected according to experiments No. 1, 2, 5 and 8 in Table 2. The calculated results are shown in Figure 12 for the resulting concentration at equilibrium. For scenarios with *D*o < 1, all drug particles dissolve, *i.e.*, pH 2 for 25 and 100 mg and pH 3 for 25 mg, reaching 100 % drug dissolution. For scenarios where *D*o >> 1, only partial drug dissolution can be achieved, which depends on the drug nanocrystal particle size distribution, as seen in the *in vivo* investigations. Thus, dissolution testing under conditions with *D*o >> 1 enables the distinction between formulations containing different drug nanocrystal particle size distributions.

**Figure 13.**
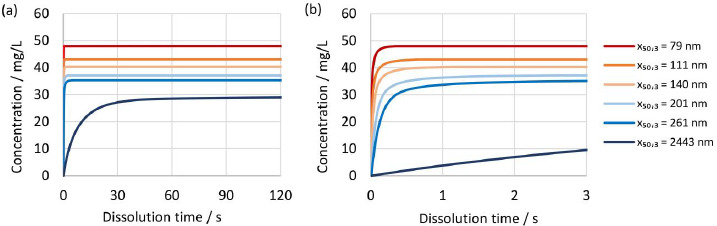
Calculated results for the evolution of concentration for *in vitro* dissolution scenarios of drug nanocrystal particle size distributions with *x*_50,3_ of 2443 nm, 261 nm, 201 nm, 140 nm, 111 nm and 79 nm according to Table 2, 100 mg dose strength in 900 mL dissolution media of pH 3 and *D*o = 4. **(a)** Calculated results for full dissolution of all scenarios. **(b)** Calculated results identical to (a) but highlighted for the initial time period.

The established numerical model was further utilized to investigate the dissolution kinetics of drug nanocrystals. Figure 13(a) and (b) show the evolution of concentration over time for different drug nanocrystal particle size distributions according to experiments No. 1 to 5 and 8 in Table 2 for constant dissolution conditions of 100 mg dose strength in 900 mL dissolution media of pH 3 with *D*o = 4. Most notably, there is a big difference between the dissolution kinetics of the coarse crystal suspension with a median particle size *x*_50,3_ of 2443 nm and all other drug nanocrystal suspensions with median particle sizes *x*_50,3_ of 258 nm or lower. Ultrafine drug nanocrystal particle sizes result in rapid dissolution kinetics and reach the equilibrium state almost instantaneously after only a few seconds. It should be highlighted that the rapid dissolution is caused by the combination of increased specific surface area and increased apparent solubility, both due to the very fine particle sizes. In addition, the concentration at equilibrium state scales with decreasing particle size distribution.

Due to the rapid dissolution kinetics, it is challenging for standard *in vitro* dissolution testing under sink conditions to discriminate between different particle size distributions containing ultrafine drug nanocrystals. Observed differences in dissolution kinetics of drug products containing drug nanocrystals of different particle sizes, must therefore be related to other functional properties of the drug product, *i.e.*, disintegration and dispersion.

**Figure 14.**
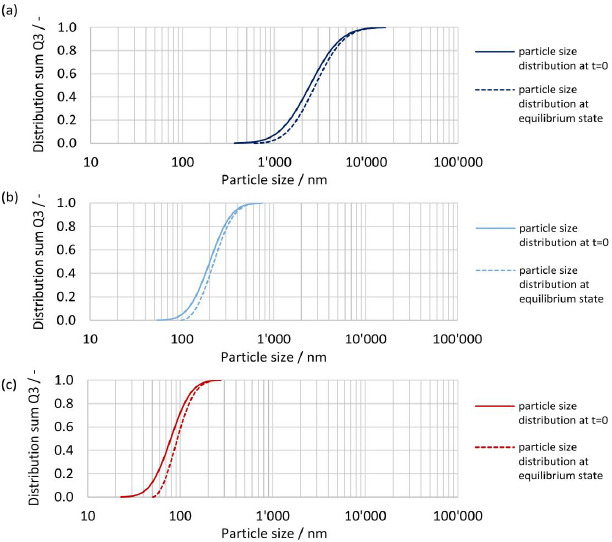
Calculated results for the evolution of nanocrystal particle size distributions for *in vitro* dissolution scenarios with 100 mg dose strength in 900 mL dissolution media of pH 3 and *D*o = 4, corresponding to Figure 12. **(a)** *x*_50,3_ = 2443 nm. **(b)** *x*_50,3_ = 201 nm. **(c)** *x*_50,3_ = 79 nm.

The initial particle size distribution at t = 0 and the resulting particle size distribution at equilibrium are shown in Figures 14(a), (b) and (c) for drug nanocrystals with median particle size *x*_50,3_ of 2443 nm, 201 nm and 79 nm, see experiment No. 1, 3 and 8 in Table 2. The biggest change in the shape of the particle size distribution is seen for the finest drug nanocrystals, where the fine fraction is notably reduced, see Figure 14(c). On the other hand, the shape of the coarsest crystal suspension remains almost unchanged, see Figure 14(a). This is caused by the relative difference in apparent solubility of the particles at the lower and upper end of the particle size distribution, which is more pronounced for smaller particle sizes. Hence, the smallest particles dissolve much faster than the coarser particles, resulting in a change in the shape of the particle size distribution. This emphasizes once more the need to consider the entire particle size distribution by a time-resolved calculation. It should be noted that the smallest still remaining particle size at the end of the dissolution correlates directly to the calculated concentration at equilibrium.

**Figure 15.**
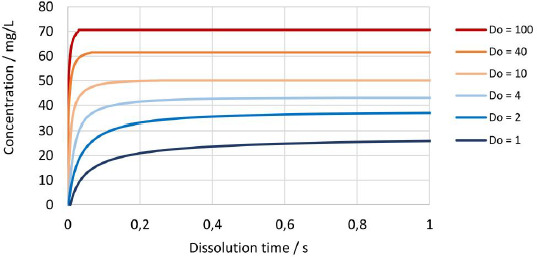
Calculated results for the evolution of concentration for *in vitro* dissolution scenarios for drug nanocrystal particle size distributions *x*_50,3_ = 111 nm according to Table 2 in 900 mL dissolution media of pH 3 with different ratios of the available solid drug to the thermodynamically soluble amount *D*o of 1, 2, 4, 10, 40 and 100.

Figure 15 shows the change in concentration over time for the drug nanocrystals with median particle size *x*_50,3_ of 111 nm at *D*o values from 1 to 100. Naturally, the dissolution kinetics is faster for higher *D*o values since the more absolute surface area is available for dissolution. In addition, an increased amount of fine fraction is present, which dissolves faster due to increased apparent solubility, which further enhances the dissolution rate and results in a notable increase of the resulting concentration at the equilibrium state.

**Figure 16.**
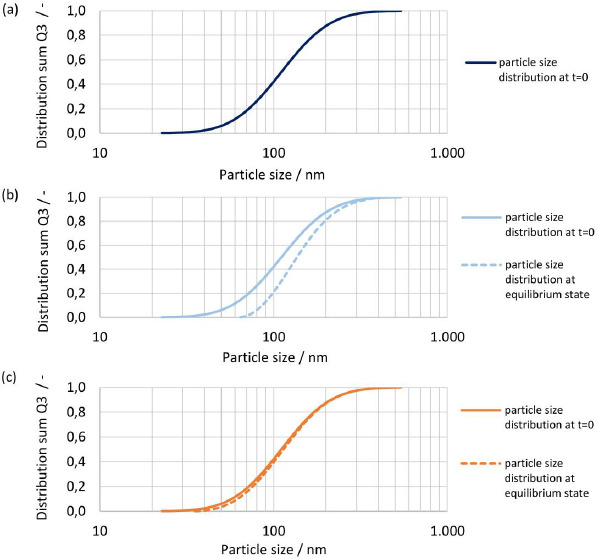
Calculated results for the evolution of nanocrystal particle size distributions for *in vitro* dissolution scenarios with initial particle size *x*_50,3_ = 111 nm in 900 mL dissolution media of pH 3 corresponding to Figure 12. **(a)** *D*o = 1. **(b)** *D*o = 4. **(c)** *D*o = 40.

The initial particle size distribution at *t* = 0 and the resulting particle size distribution at equilibrium are shown in Figures 16(a), (b) and (c) for *D*o numbers of 1, 4 and 40 for drug nanocrystals with median particle size *x*_50,3_ of 111 nm. For *D*o = 1 all material dissolved, hence no resulting particle size distribution is shown, see Figure 16(a). For increasing *D*o, the change in the shape of the particle size distribution decreases. While a notable change is seen with *D*o = 4, barely any change is observed with *D*o = 40. Similarly, as observed before, the change of shape of the particle size distribution is the biggest for the fine fraction due to increased apparent solubility and the smallest still remaining particle size at the end of the dissolution correlates directly to the apparent solubility at the equilibrium state.

## Conclusions

Nanocrystals with different particle size distributions were manufactured for a drug compound with constant formulation composition utilizing wet media milling technology with an industrially applicable process design. The finest manufactured size distribution resulted in a particle size *x*_10_ / *x*_50_ / *x*_90_ of 47 / 79 / 133 nm. High magnification microscopy even showed ultrafine nanocrystals with an almost sphere-like shape of about 20 nm. The manufactured nanocrystals are considered crystalline, however, for particles below 200 nm an increasing amount of amorphous content with decreasing particle size becomes detectable and quantifiable with reflection X-ray powder diffraction up to a maximum of about 20 % amorphous content for the finest drug nanocrystal particle size distribution. This amorphous content is considered an intrinsic property and was explained by a simple model estimating the intrinsic surface amorphicity of the nanocrystals due to the high specific surface area and related small particle size/high curvature. The experimentally determined apparent solubility of the different nanocrystal particle size distributions showed consistent results at pH 2, pH 3 and pH 4.5, with a notable increase of the apparent solubility from coarse micron-sized crystal to ultrafine nanocrystals particle size distribution. The highest supersaturation was identified for the finest nanocrystal size distribution with almost a factor of 2 compared to the thermodynamic solubility of the coarse drug compound. An increasing dose number (*D*o) notably influences the measured apparent solubility due to the increasing amount of fine fraction available for dissolution. Interestingly, the results obtained for apparent solubility against drug particle size coincide in trend with the results obtained on the amorphous content associated with the intrinsic amorphous surface layer.

The theoretical apparent solubility calculated by the established numerical model for drug nanocrystal dissolution in comparison to the experimentally determined apparent solubility showed conclusive results. The assumption of monodisperse particle sizes for calculation failed to describe the experimentally determined apparent solubility for the polydisperse nanocrystal particle size distributions by notably underpredicting the experimental values, as well as the inability to describe the influence of the dose number. The consideration of the entire particle size distribution and its evolution over time is essential to reliably and accurately describe the dissolution behavior of nanocrystals. The developed numerical model according to the Noyes-Whitney / Nernst-Brunner and Ostwald-Freundlich equations, including the plausible value for interfacial energy of 30 mJ/m^2^ is conclusive and is deemed to be accurate up to *D*o < 18 with a reasonable error of < 10 % between experimental and calculated apparent solubility for the investigated drug stabilizer system.

The presented numerical model can be utilized to describe the dissolution behavior of other nanoparticulate compounds. To adjust the model to a compound of interest, the compound-specific properties density, molar volume and thermodynamic solubility in the medium of interest as well as the sample-specific particle size distribution, are required. In addition, a few experimental data points for the apparent solubility of samples with different particle size distributions are needed to determine the interfacial energy by fitting.

*In silico* dissolution studies were conducted with the numerical model for selected *in vivo* and *in vitro* scenarios to simulate the complex interplay between drug nanocrystal particle size distribution, drug amount, dissolution media and volume, and resulting temporal progression of concentration during dissolution up to equilibrium state as well as remaining drug particle size distribution, if any. It has been shown that ultrafine drug nanocrystal particle sizes result in rapid dissolution kinetics, caused by increased specific surface area and increased apparent solubility, and in reaching an almost instantaneously equilibrium state after only a few seconds. The highest equilibrium concentrations are obtained for *D*o >> 1 and ultrafine drug nanocrystal particle size distributions. The numerical model offers the possibility to easily calculate and flexibly adjust the scenario to any *in vivo* and *in vitro* application of interest. The calculated results illustrate the impact of the configurable formulation variables nanocrystal particle size distribution and dose strength on the obtained dissolution kinetics and supersaturation level in a given dissolution medium and volume. This is of vital importance for industrial product and dissolution method development since increased dissolution kinetics and supersaturation are generally accepted contributing factors for potential oral bioavailability improvement.

## Figures and Tables

**Table 1.** Input parameters for the established numerical model.

Parameter	Value	Unit
Drug molar volume, *V*_m_	3.72·10^-4^	m^3^/mol
Dynamic viscosity of dissolution media, *η*	1	mPa s
Interfacial energy between solid and liquid, *σ*_sl_	30	mJ/m^2^
Solid drug density, *ρ*	1364	kg/m^3^
Thermodynamic solubility, *s*_0,pH3_	28	mg/L

**Table 2.** Volume-based (Q_3_) particle size distribution percentiles (*x*_10,3_, *x*_50,3_ and *x*_90,3_) and standard deviation (σ) for approximated dimensionless log-normal distributions used as input for the numerical model and deviation (in brackets) to experimental particle size distributions determined by laser light diffraction (LLD) for experiment No. 1 and by photon correlation spectroscopy (PCS) for experiments No. 2 to 8.

Experiment No.	Particle size *x*_10,3_ / nm	Particle size *x*_50,3_ / nm	Particle size *x*_90,3_ / nm	Standard deviation *σ* / -
1	1116 (-0.4%)	2443 (-1.9%)	5344 (+0.1%)	0.611
2	152 (-3.4%)	261 (+1.0%)	449 (-3.6%)	0.422
3	116 (-2.2%)	201 (+0.0%)	348 (-0.9%)	0.427
4	78 (-6.0%)	140 (+1.4%)	252 (-3.2%)	0.456
5	57 (-5.9%)	111 (+2.2%)	213 (-3.5%)	0.513
6	53 (-7.2%)	93 (+2.5%)	162 (-7.1%)	0.432
7	53 (-6.4%)	90 (+2.8%)	150 (-7.0%)	0.404
8	47 (-4.3%)	79 (+1.1%)	133 (-3.9%)	0.402

**Table 3.** Estimated relative amount of the amorphous content (AM) for different particle sizes (*x*) and a different number of layers of unit cells (*Z*).

Particle size / nm	Amorphous content / %		
	*Z*=1	*Z*=2	*Z*=3
20	28.5	51.0	68.1
50	12.2	23.3	33.4
100	6.2	12.2	17.8
200	3.1	6.2	9.2
1000	0.6	1.3	1.9
3000	0.2	0.4	0.6
100000	0.01	0.01	0.02

**Table 4.** Nanocrystal particle size *x*_50,3_ for experiments No.1 and 6 to 8 and calculated crystallite size from transmission XRPD peak data at 13.7° 2*ϴ*.

Experiment No.	Particle size *x*_50,3_ / nm	Intensity / cps	Crystallite size / Å
1	2443	2114	1050
6	93	618	320
7	90	563	285
8	79	516	325

**Table 5.** Calculated crystalline content (*w*_cryst,XRPD_) and amorphous content (*w*_amorph,XRPD_) by reflection XRPD for experiment No.1, 5 and 8 and calculated amorphous content for a single layer of unit cell and related median particle size *x*_50,3_ (*w*_amorph, Z=1_).

Experiment No.	Particle size *x*_50,3_ / nm	*w*_cryst,XRPD_ / %	*w*_amorph,XRPD_ / %	*w*_amorph__Z=1_ / %
1	2443	92 ±1	8	0.3
5	111	77 ±3	23	5.6
8	79	80 ±3	20	7.8

**Table 6.** Estimated relative amount of the amorphous content (*w*_amorph,Z_) for experiments No. 5 and 8 considering the whole particle size distribution and a different number of layers of unit cells (*Z*).

Experiment No.	Particle size *x*_50,3_ / nm	Amorphous content *w*_amorph,Z_ / %		
		Z=1	Z=2	Z=3
5	111	6.3	12.3	17.9
8	79	9.0	17.4	25.1

**Table 7.** Experimental results for apparent solubility (*s*_app,exp_) for polydisperse particle size distributions according to Table 2 as determined by high-pressure liquid chromatography (HPLC) in dissolution media of pH 3 at 22 °C.

Experiment No.	Solids amount *m* / g	Dose number *D*o / -	Particle size *x*_50,3_ / nm	Solubility in pH 3 *s*_app,exp_ / mg/L
1	0.25	8.9	2443	30.3
2	0.25	8.9	261	34.9
3	0.25	8.9	201	39.8
4	0.25	8.9	140	48.1
5	0.25	8.9	111	49.9
6	0.25	8.9	93	53.9
7	0.25	8.9	90	52.6
8	0.25	8.9	79	55.7
9	0.075	2.7	111	41.6
10	0.125	4.5	111	47.2
11	0.25	8.9	111	49.9
12	0.5	18	111	51.3
13	1.25	45	111	51.9
14	2.5	89	111	52.1
15	5	179	111	52.5

## Abbreviations

α, β, γ: angles of the unit cell’s axis
δ: diffusion boundary layer thickness
η: dynamic viscosity of the liquid
ρ: density
σ: standard deviation
σ_sl_: interfacial energy between solid and liquid phase
a, b, c: length of the unit cells axis
A: surface area
AM: amount of amorphous surface layer
c_l_: concentration in the liquid phase
cps: counts per second
D: diffusion coefficient
D_cell_: diameter of the crystallographic unit cell
Do: Dose number, ratio of available solids to thermodynamically soluble amount
i: index, number of particle class
j: index, number of iterations
k_B_: Boltzmann constant
m: mass
NIST: National Institute of Standards and Technology
r: particle radius
R: universal gas constant
R_0_: hydrodynamic radius of diffusing particles
s: solubility
s_0_: thermodynamic solubility
s_app_: apparent solubility
s_app,exp_: experimentally determined apparent solubilitys_app,th_ theoretical, calculated apparent solubility
SRM: Standard reference material
t: time
Δt: time step
T: Temperature
V_cell_: volume of one unit cell
V_l_: Volume of the liquid
V_m_: molar volume of dissolving compound
w_amorph,XRPD_: calculated amorphous content by XRPD measurement
w_cryst,XRPD_: calculated crystalline content by XRPD measurement
w_amorph,Z_: calculated amorphous content for number of layers of unit cells by model
x: particle size
y: number of particle size classes that can further dissolve
Z: depths of amorphous surface layer, expressed in layers of unit cells

## References

[1] Rabinow B.R.. Nanosuspensions in drug delivery. Nature Reviews Drug Discovery 3 (2004) 785-795. https://doi.org/10.1038/nrd1494. 10.1038/nrd1494 15340388

[2] Liversidge E.M. Liversidge G.G. Cooper E.R.. Nanosizing: a formulation approach for poorly-water-soluble compounds. European Journal of Pharmaceutical Sciences 18 (2003) 113-120. https://-doi.org/10.1016/S0928-0987(02)00251-8. 10.1016/S0928-0987(02)00251-8 12594003

[3] Kesisoglou F. Panmai S. Wu Y.. Nanosizing – Oral formulation development and biopharmaceutical evaluation. Advanced Drug Delivery Reviews 59 (2007) 631-644. https://doi.org/10.1016/-j.addr.2007.05.003. 10.1016/-j.addr.2007.05.003 17601629

[4] Wong J. Brugger A. Khare A. Chaubal M. Papadopoulos P. Rabinow B. Kipp J. Ning J.. Suspensions for intravenous (IV) injection: A review of development, preclinical and clinical aspects. Advanced Drug Delivery Reviews 60 (2008) 939-954. https://doi.org/10.1016/j.addr.2007.11.008. 10.1016/j.addr.2007.11.008 18343527

[5] Liversidge E.M. Liversidge G.G.. Nanosizing for oral and parenteral drug delivery: A perspective on formulating poorly-water soluble compounds using wet media milling technology. Advanced Drug Delivery Reviews 63 (2011) 427-440. https://doi.org/10.1016/j.addr.2010.12.007. 10.1016/j.addr.2010.12.007 21223990

[6] Müller R.H. Gohla S. Keck C.M.. State of the art of nanocrystals – Special features, production, nanotoxicology aspects and intracellular delivery. European Journal of Pharmaceutics and Biopharmaceutics 78 (2011) 1-9. https://doi.org/10.1016/j.ejpb.2011.01.007. 10.1016/j.ejpb.2011.01.007 21266197

[7] Müller R.H. Keck C.M.. Twenty years of drug nanocrystals: Where are we, and where do we go? European Journal of Pharmaceutics and Biopharmaceutics 80 (2012) 1-3. https://doi.org/10.1016/-j.ejpb.2011.09.012. 10.1016/-j.ejpb.2011.09.012 21971369

[8] Shah D.A. Murdande S.B. Dave R.H.. A review: Pharmaceutical and pharmacokinetic aspect of nanocrystalline suspensions. Journal of Pharmaceutical Sciences 105 (2016) 10-24. http://-dx.doi.org/10.1002/jps.24694. 10.1002/jps.24694 26580860

[9] Chen M.L. John M. Lee S.L. Tyner K.M.. Development considerations for nanocrystal drug products. The AAPS Journal 19 (2017) 642-651. https://doi.org/10.1208/s12248-017-0064-x. 10.1208/s12248-017-0064-x 28281194

[10] Peltonen L. Strachan C.J.. Degrees of order: A comparison of nanocrystal and amorphous solids for poorly soluble drugs. International Journal of Pharmaceutics 586 (2020) 119492. https://doi.org/-10.1016/j.ijpharm.2020.119492. 10.1016/j.ijpharm.2020.119492 32505579

[11] Dahlgren D. Sjögren E. Lennernäs H.. Intestinal absorption of BCS class II drugs administered as nanoparticles: A review based on in vivo data from intestinal perfusion models. ADMET & DMPK 8 (2020) 375-390. https://dx.doi.org/10.5599/admet.881. 10.5599/admet.881 35300192 PMC8915587

[12] Ma Z. Zhang H. Wang Y. Tang X.. Development and evaluation of intramuscularly administered nano/microcrystal suspension. Expert Opinion on Drug Delivery 16 (2019) 347-361. https://doi.org/-10.1080/17425247.2019.1588248. 10.1080/17425247.2019.1588248 30827123

[13] Jain R. Meyer J. Wehr A. Rege B. von Moltke L. Weiden P.J.. Size matters: the importance of particle size in a newly developed injectable formulation for the treatment of schizophrenia. CNS Spectrums 25 (2020) 323-330. https://doi.org/10.1017/S1092852919000816. 10.1017/S1092852919000816 31111801

[14] Smith W.C. Bae J. Zhang Y. Qin B. Wang Y. Kozak D. Ashraf M. Xu X.. Impact of particle flocculation on the dissolution and bioavailability of injectable suspensions. International Journal of Pharmaceutics 604 (2021) 120767. https://doi.org/10.1016/j.ijpharm.2021.120767. 10.1016/j.ijpharm.2021.120767 34087414

[15] Merisko-Liversidge E. Liversidge G.G.. Nanosizing for oral and parenteral drug delivery: A perspective on formulating poorly-water soluble compounds using wet media milling technology. Advanced Drug Delivery Reviews 63 (2011) 427-440. https://doi.org/10.1016/j.addr.2010.12.007. 10.1016/j.addr.2010.12.007 21223990

[16] Möschwitzer J.P.. Drug nanocrystals in the commercial pharmaceutical development process. International Journal of Pharmaceutics 453 (2013) 142-156. https://doi.org/10.1016/-j.ijpharm.2012.09.034. 10.1016/-j.ijpharm.2012.09.034 23000841

[17] Li M. Azad M. Dave R. Bilgili E.. Nanomilling of drugs for bioavailability enhancement: A holistic formulation-process perspective. Pharmaceutics 8 (2016) 17. https://doi.org/10.3390/-pharmaceutics8020017. 10.3390/-pharmaceutics8020017 PMC4932480 27213434

[18] Malamatari M. Taylor K.M.G. Malamataris S. Douroumis D. Kachrimanis K.. Pharmaceutical nanocrystals: Production by wet milling and applications. Drug Discovery Today 23 (2018) 534-547. https://doi.org/10.1016/j.drudis.2018.01.016. 10.1016/j.drudis.2018.01.016 29326082

[19] Peltonen L. Hirvonen J.. Drug nanocrystals – Versatile option for formulation of poorly soluble materials. International Journal of Pharmaceutics 537 (2018) 73-83. https://doi.org/10.1016/-j.ijpharm.2017.12.005. 10.1016/-j.ijpharm.2017.12.005 29262301

[20] Tanaka Y. Inkyo M. Yumoto R. Nagai J. Takano M. Nagata S.. Nanoparticulation of probucol, a poorly water-soluble drug, using a novel wet-milling process to improve *in vitro* dissolution and *in vivo* oral absorption. Drug Development and Industrial Pharmacy 38 (2012) 1015-1023. https://-doi.org/10.3109/03639045.2011.637051. 10.3109/03639045.2011.637051 22118063

[21] Li M. Yaragudi N. Afolabi A. Dave R. Bilgili E.. Sub-100 nm drug particle suspensions prepared via wet milling with low bead contamination through novel process intensification. Chemical Engineering Science 130 (2015) 207.220. https://doi.org/10.1016/j.ces.2015.03.020. 10.1016/j.ces.2015.03.020

[22] Juenemann D. Jantratid E. Wagner C. Reppas C. Vertzoni M. Dressman J.B.. Biorelevant in vitro dissolution testing of products containing micronized or nanosized fenofibrate with a view to predicting plasma profiles. European Journal of Pharmaceutics and Biopharmaceutics 77 (2011) 257-264. https://doi.org/10.1016/j.ejpb.2010.10.012. 10.1016/j.ejpb.2010.10.012 21074611

[23] Hens B. Brouwers J. Corsetti M. Augustijns P.. Gastrointestinal behavior of nano- and microsized fenofibrate: In vivo evaluation in man and in vitro simulation by assessment of the permeation potential. European Journal of Pharmaceutical Sciences 77 (2015) 40-47. https://doi.org/10.1016/-j.ejps.2015.05.023. 10.1016/-j.ejps.2015.05.023 26004010

[24] Roos C. Dahlgren D. Berg S. Westergren J. Abrahamsson B. Tannergren C. Sjögren E. Lennernäs H.. In vivo mechanisms of intestinal drug absorption from aprepitant nanoformulations. Molecular Pharmaceutics 14 (2017) 4233-4242. https://doi.org/10.1021/-acs.molpharmaceut.7b00294. 10.1021/-acs.molpharmaceut.7b00294 28737398

[25] Roos C. Westergren J. Dahlgren D. Lennernäs H. Sjögren E.. Mechanistic modelling of intestinal drug absorption – The in vivo effects of nanoparticles, hydrodynamics, and colloidal structures. European Journal of Pharmaceutics and Biopharmaceutics 133 (2018) 70-76. https://doi.org/-10.1016/j.ejpb.2018.10.006. 10.1016/j.ejpb.2018.10.006 30300720

[26] Litou C. Patel N. Turner D.B. Kostewicza E. Kuentz M. Box K.J. Dressman J.. Combining biorelevant in vitro and in silico tools to simulate and better understand the in vivo performance of a nano-sized formulation of aprepitant in the fasted and fed states. European Journal of Pharmaceutical Sciences 138 (2019) 105031. https://doi.org/10.1016/j.ejps.2019.105031. 10.1016/j.ejps.2019.105031 31386891

[27] Imono M. Uchiyama H. Yoshida S. Miyazaki S. Tamura N. Tsutsumimoto H. Kadota K. Tozuka Y.. The elucidation of key factors for oral absorption enhancement of nanocrystal formulations: In vitro–in vivo correlation of nanocrystals. European Journal of Pharmaceutics and Biopharmaceutics 146 (2020) 84-92. https://doi.org/10.1016/j.ejpb.2019.12.002. 10.1016/j.ejpb.2019.12.002 31816392

[28] Guo M. Wei M. Li W. Guo M. Guo. C. Ma M. Wang Y. Yang Z. Li M. Fu Q. Yang L. He Z.. Impacts of particle shapes on the oral delivery of drug nanocrystals: Mucus permeation, transepithelial transport and bioavailability. Journal of Controlled Release 307 (2019) 64-75. https://doi.org/10.1016/j.jconrel.2019.06.015. 10.1016/j.jconrel.2019.06.015 31207275

[29] Abrahamsson B. McAllister M. Augustijns P. Zane P. Butler J. Holm R. Langguth P. Lindahl A. Müllertz A. Pepin X. Rostami-Hodjegan A. Sjögren E. Berntsson M. Lennernäs H.. Six years of progress in the oral biopharmaceutics area – A summary from the IMI OrBiTo project. European Journal of Pharmaceutics and Biopharmaceutics 152 (2020) 236-247. https://doi.org/10.1016/-j.ejpb.2020.05.008. 10.1016/-j.ejpb.2020.05.008 32446960

[30] Palmelund H. Eriksen J.B. Bauer-Brandl A. Rantanen J. Löbmann K.. Enabling formulations of aprepitant: in vitro and in vivo comparison of nanocrystalline, amorphous and deep eutectic solvent based formulations. International Journal of Pharmaceutics: X 3 (2021) 100083. https://-doi.org/10.1016/j.ijpx.2021.100083. 10.1016/j.ijpx.2021.100083 PMC8193149 34151250

[31] Liu P. De Wulf O. Laru J. Heikkilä T. Van Veen B. Kiesvaara J. Hirvonen J. Peltonen L. Laaksonen T.. Dissolution studies of poorly soluble drug nanosuspensions in non-sink conditions. AAPS PharmSciTech 14 (2013) 748-756. https://doi.org/10.1208/s12249-013-9960-2. 10.1208/s12249-013-9960-2 23615772 PMC3666001

[32] Braig V. Konnerth C. Peukert W Lee G.. Enhanced dissolution of naproxen from pure-drug, crystalline nanoparticles: A case study formulated into spray-dried granules and compressed tablets. International Journal of Pharmaceutics 554 (2019) 54-60. https://doi.org/10.1016/-j.ijpharm.2018.09.069. 10.1016/-j.ijpharm.2018.09.069 30278257

[33] Sironi D. Rosenberg J. Bauer-Brandl A. Brandl M.. Dynamic dissolution-/permeation-testing of nano- and microparticle formulations of fenofibrate. European Journal of Pharmaceutical Sciences 96 (2017) 20-27. https://doi.org/10.1016/j.ejps.2016.09.001. 10.1016/j.ejps.2016.09.001 27597143

[34] Tsinman K. Tsinman O. Lingamaneni R. Zhu S. Riebesehl B. Grandeury A. Juhnke M. Van Eerdenbrugh B.. Ranking itraconazole formulations based on the flux through artificial lipophilic membrane. Pharmaceutical Research 35 (2018) 161. https://doi.org/10.1007/s11095-018-2440-3. 10.1007/s11095-018-2440-3 29926245

[35] Li J. Li L.B. Nessah N. Huang Y. Hidalgo C. Owen A. Hidalgo I.J.. Simultaneous analysis of dissolution and permeation profiles of nanosized and microsized formulations of indomethacin using the in vitro dissolution absorption system 2. Journal of Pharmaceutical Sciences 108 (2019) 2334-2340. https://doi.org/10.1016/j.xphs.2019.01.032. 10.1016/j.xphs.2019.01.032 30776382

[36] Imono M. Uchiyama H. Ueda H. Kadota K. Tozuka Y.. In-situ dissolution and permeation studies of nanocrystal formulations with second-derivative UV spectroscopy. International Journal of Pharmaceutics 558 (2019) 242249. https://doi.org/10.1016/j.ijpharm.2018.12.086. 10.1016/j.ijpharm.2018.12.086 30654061

[37] Arce F.A. Setiawan N. Campbell H.R. Lu X. Nethercott M.J. Bummer P. Su Y. Marsac P.J.. Toward developing discriminating dissolution methods for formulations containing nanoparticulates in solution: The impact of particle drift and drug activity in solution. Molecular Pharmaceutics 17 (2020) 4125-4140. https://doi.org/10.1021/acs.molpharmaceut.0c00599. 10.1021/acs.molpharmaceut.0c00599 32965123

[38] Tian H. Qin Z. Wang G. Yu X. Chen J. Lin Z. Du S. Yin H. Zou H. Liu T.. Consideration of the dissolution media for drug nanocrystal evaluation. Powder Technology 392 (2021) 179-190. https://doi.org/10.1016/j.powtec.2021.07.016. 10.1016/j.powtec.2021.07.016

[39] Elkhabaz A. Moseson D.E. Brouwers J. Augustijns P. Taylor L.S.. Interplay of supersaturation and solubilization: Lack of correlation between concentration-based supersaturation measurements and membrane transport rates in simulated and aspirated human fluids. Molecular Pharmaceutics 16 (2019) 5042-5053. https://doi.org/10.1021/acs.molpharmaceut.9b00956. 10.1021/acs.molpharmaceut.9b00956 31638397

[40] Jünemann D. Dressman J.. Analytical methods for dissolution testing of nanosized drugs. Journal of Pharmacy and Pharmacology 64 (2012) 931-943. https://doi.org/10.1111/j.2042-7158.2012.01520.x. 10.1111/j.2042-7158.2012.01520.x 22686341

[41] Anhalt K. Geissler S. Harms M. Weigandt M. Fricker G.. Development of a new method to assess nanocrystal dissolution based on light scattering. Pharmaceutical Research 29 (2012) 2887-2901. https://doi.org/10.1007/s11095-012-0795-4. 10.1007/s11095-012-0795-4 22688901

[42] Van Eerdenbrugh B. Alonzo D.E. Taylor L.S.. Influence of particle size on the ultraviolet spectrum of particulate-containing solutions: Implications for in-situ concentration monitoring using UV/Vis fiber-optic probes. Pharmaceutical Research 28 (2011) 1643-1652. https://doi.org/10.1007/s11095-011-0399-4. 10.1007/s11095-011-0399-4 21374101

[43] Van Eerdenbrugh B. Vermant J. Martens J.A. Froyen L. Van Humbeeck J. Van den Mooter G. Augustijns P.. Solubility increases associated with crystalline drug nanoparticles: Methodologies and significance. Molecular Pharmaceutics 7 (2010) 1858-1870. https://doi.org/10.1021/mp100209b. 10.1021/mp100209b 20822111

[44] Murdande S.B. Shah D.A. Dave R.H.. Impact of nanosizing on solubility and dissolution rate of poorly soluble pharmaceuticals. Journal of Pharmaceutical Sciences 104 (2015) 2094-2102. https://-doi.org/10.1002/jps.24426. 10.1002/jps.24426 25821105

[45] Colombo M. Staufenbiel S. Rühl E. Bodmeier R.. In situ determination of the saturation solubility of nanocrystals of poorly soluble drugs for dermal application. International Journal of Pharmaceutics 521 (2017) 156-166. https://doi.org/10.1016/j.ijpharm.2017.02.030. 10.1016/j.ijpharm.2017.02.030 28223247

[46] Colombo M. Minussi C. Orthmann S. Staufenbiel S. Bodmeier R.. Preparation of amorphous indomethacin nanoparticles by aqueous wet bead milling and in situ measurement of their increased saturation solubility. European Journal of Pharmaceutics and Biopharmaceutics 125 (2018) 159-168. https://doi.org/10.1016/j.ejpb.2018.01.013. 10.1016/j.ejpb.2018.01.013 29371046

[47] Lu A.T.K. Frisella M.E. Johnson K.C.. Dissolution modeling: Factors affecting the dissolution rates of polydisperse powders. Pharmceutical Research 10 (1993) 1308-1314. https://doi.org/10.1023/-A:1018917729477. 10.1023/-A:1018917729477 8234168

[48] Wang J. Flanagan D.R.. General solution for diffusion-controlled dissolution of spherical particles. 1. Theory. Journal of Pharmaceutical Sciences 88 (1999) 731-738. https://doi.org/10.1021/js980236p. 10.1021/js980236p 10393573

[49] Wang J. Flanagan D.R.. General solution for diffusion-controlled dissolution of spherical particles. 2. Evaluation of experimental data. Journal of Pharmaceutical Sciences 91 (2002) 534-542. https://doi.org/10.1002/jps.10039. 10.1002/jps.10039 11835211

[50] Wang Y. Abrahamsson B. Lindfors L. Brasseur J.G.. Analysis of diffusion-controlled dissolution from polydisperse collections of drug particles with an assessed mathematical model. Journal of Pharmaceutical Sciences 104 (2015) 2998-3017. https://doi.org/10.1002/jps.24472. 10.1002/jps.24472 25989144

[51] Abrami M. Grassi L. di Vittorio R. Hasa D. Perissutti B. Voinovich D. Grassi G. Colombo I. Grassi M.. Dissolution of an ensemble of differently shaped poly-dispersed drug particles undergoing solubility reduction: mathematical modelling. ADMET & DMPK 8 (2020) 297-313. http://dx.doi.org/-10.5599/admet.841. 10.5599/admet.841 35300307 PMC8915606

[52] Gao Y. Glennon B. He Y. Donnellan P.. Dissolution kinetics of a BCS Class II active pharmaceutical ingredient: Diffusion-based model validation and prediction. ACS Omega 6 (2021) 8056-8067. https://doi.org/10.1021/acsomega.0c05558. 10.1021/acsomega.0c05558 33817465 PMC8014923

[53] Ely D.R. García R.E. Thommes M.. Ostwald–Freundlich diffusion-limited dissolution kinetics of nanoparticles. Powder Technology 257 (2014) 120-123. https://doi.org/10.1016/-j.powtec.2014.01.095. 10.1016/-j.powtec.2014.01.095

[54] Johnson K.C.. Comparison of methods for predicting dissolution and the theoretical implications of particle-size-dependent solubility. Journal of Pharmaceutical Sciences 101 (2012) 681-689. https://-doi.org/10.1002/jps.22778. 10.1002/jps.22778 21989679

[55] Parks C. Koswara A. Tung H.H. Nere N.K. Bordawekar S. Nagy Z.K. Ramkrishna D.. Nanocrystal dissolution kinetics and solubility increase prediction from molecular dynamics: The case of α‑, β‑, and γ‑Glycine. Molecular Pharmaceutics 14 (2017) 1023-1032. https://doi.org/10.1021/-acs.molpharmaceut.6b00882. 10.1021/-acs.molpharmaceut.6b00882 28271901

[56] Owens D.K. Wendt R.C.. Estimation of the surface free energy of polymers. Journal of Applied Polymer Science 13 (1969) 1741-1747. https://doi.org/10.1002/app.1969.070130815. 10.1002/app.1969.070130815

[57] Kaelble D.H.. Dispersion-polar surface tension properties of organic solids. The Journal of Adhesion 66 (1970) 66-81. https://doi.org/10.1080/0021846708544582. 10.1080/0021846708544582

[58] Dorris G.M. Gray D.G.. Adsorption of n-alkanes at zero surface coverage on cellulose paper and wood fibers. Journal of Colloid and Interface Science 77 (1980) 353-362. https://doi.org/10.1016/0021-9797(80)90304-5. 10.1016/0021-9797(80)90304-5

[59] ISO 13320:2020: Particle size analysis – Laser diffraction methods (2020).

[60] ISO 22412:2008: Particle size analysis – Dynamic light scattering (DLS) (2008).

[61] ISO 489:2022: Plastics – Determination of refractive index (2022).

[62] Noyes A.A. Whitney W.R., The rate of solution of solid substances in their own solutions. Journal of the American Chemical Society 19 (1897) 930-934. https://doi.org/10.1021/ja02086a003. 10.1021/ja02086a003

[63] Nernst W.. Theorie der Reaktionsgeschwindigkeit in heterogenen Systemen. Zeitschrift für Physikalische Chemie 47 (1904) 52-55. https://doi.org/10.1515/zpch-1904-4704. 10.1515/zpch-1904-4704

[64] Prandtl L.. Verhandlungen des dritten internationalen Mathematiker-Kongresses in Heidelberg 1904 vom 8. bis 14. August 1904. B.G. Teubner, Leipzig, Germany, 1905, p. 484.

[65] Bisrat M. Nyström C.. Physicochemical aspects of drug release. VIII. The relation between particle size and surface specific dissolution rate in agitated suspensions. International Journal of Pharmaceutics 47 (1988) 223-231. https://doi.org/10.1016/0378-5173(88)90235-9. 10.1016/0378-5173(88)90235-9

[66] Niebergall P.J. Milosovich G. Goyan J.E.. Dissolution rate studies. II. Dissolution of particles under conditions of rapid agitation. Journal of Pharmaceutical Sciences 52 (1963) 236-241. https://doi.org/-10.1002/jps.2600520310. 10.1002/jps.2600520310 13938476

[67] Higuchi W.I. Hiestand E.N.. Dissolution rates of finely divided drug powders. I. Effect of a distribution of particle sizes in a diffusion-controlled process. Journal of Pharmaceutical Sciences 52 (1963) 67-71. https://doi.org/10.1002/jps.2600520114. 10.1002/jps.2600520114 13954443

[68] Hintz R.J. Johnson K.C.. The effect of particle size distribution on dissolution rate and oral absorption. International Journal of Pharmaceutics 51 (1989) 9-17. https://doi.org/10.1016/0378-5173(89)90069-0. 10.1016/0378-5173(89)90069-0

[69] Galli C.. Experimental determination of the diffusion boundary layer width of micron and submicron particles. International Journal of Pharmaceutics 313 (2006) 114-122. https://doi.org/10.1016/-j.ijpharm.2006.01.030. 10.1016/-j.ijpharm.2006.01.030 16529883

[70] Sugano K.. Theoretical comparison of hydrodynamic diffusion layer models used for dissolution simulation in drug discovery and development. International Journal of Pharmaceutics 363 (2008) 73-77. https://doi.org/10.1016/j.ijpharm.2008.07.002. 10.1016/j.ijpharm.2008.07.002 18675893

[71] Lindfors L. Jonsson M. Weibull E. Brasseur J.G. Abrahamson B.. Hydrodynamic effects on drug dissolution and deaggregation in the small intestine – A study with Felodipine as a model drug. Journal of Pharmaceutical Sciences 104 (2015) 2969-2976. https://doi.org/10.1002/jps.24487. 10.1002/jps.24487 25980801

[72] Knapp L.F.. The solubility of small particles and the stability of colloids. Transactions of the Faraday Society 17 (1922) 457-465. https://doi.org/10.1039/TF9221700457. 10.1039/TF9221700457

[73] Oh D.M. Curl R.L. Amidon G.L.. Estimating the fraction dose absorbed from suspensions of poorly soluble compounds in humans: A mathematical model. Pharmaceutical Research 10 (1993) 264-270. https://doi.org/10.1023/A:1018947113238. 10.1023/A:1018947113238 8456075

[74] Yu L.X. Lipka E. Crison J.R. Amidon G.L.. Transport approaches to the biopharmaceutical design of oral drug delivery systems: prediction of intestinal absorption. Advanced Drug Delivery Reviews 19 (1996) 359-376. https://doi.org/10.1016/0169-409X(96)00009-9. 10.1016/0169-409X(96)00009-9 11540095

[75] Butler J.M. Dressman J.B.. The developability classification system: Application of biopharmaceutics concepts to formulation development. Journal of Pharmaceutical Sciences 99 (2010) 4940-4954. https://doi.org/10.1002/jps.22217. 10.1002/jps.22217 20821390

[76] Benet L.Z. Broccatelli F. Oprea T.I.. BDDCS applied to over 900 drugs. The AAPS Journal 13 (2011) 519-547. https://doi.org/10.1208/s12248-011-9290-9. 10.1208/s12248-011-9290-9 21818695 PMC3231854

[77] Wuelfing W.P. Daublain P. Kesisoglou F. Templeton A. McGregor C.. Preclinical dose number and its application in understanding drug absorption risk and formulation design for preclinical species. Molecular Pharmaceutics 12 (2015) 1031-1039. https://doi.org/10.1021/mp500504q. 10.1021/mp500504q 25671350

[78] Sugano K. Terada K.. Rate- and extent-limiting factors of oral drug absorption: Theory and applications. Journal of Pharmaceutical Sciences 104 (2015) 2777-2788. https://doi.org/10.1002/-jps.24391. 10.1002/-jps.24391 25712830

[79] Rosenberger J. Butler J. Dressman J.. A refined developability classification system. Journal of Pharmaceutical Sciences 107 (2018) 2020-2032. https://doi.org/10.1016/j.xphs.2018.03.030. 10.1016/j.xphs.2018.03.030 29665381

[80] Wuelfing W.P. El Marrouni A. Lipert M.P. Daublain P. Kesisoglou F. Converso A. Templeton A.C.. Dose number as a tool to guide lead optimization for orally bioavailable compounds in drug discovery. Journal of Medicinal Chemistry 65 (2022) 1685-1694. https://doi.org/10.1021/-acs.jmedchem.1c01687. 10.1021/-acs.jmedchem.1c01687 35060378

[81] Konnerth C. Flach F. Breitung-Faes S. Damm C. Schmidt J. Kwade A. Peukert W.. Impact of stressing conditions and polymer–surfactant interactions on product characteristics of organic nanoparticles produced by media milling. Powder Technology 294 (2016) 71-79. https://-doi.org/10.1016/j.powtec.2016.02.016. 10.1016/j.powtec.2016.02.016

[82] Peltonen L. Strachan C.J.. Degrees of order: A comparison of nanocrystal and amorphous solids for poorly soluble drugs. International Journal of Pharmaceutics 586 (2020) 119492. https://doi.org/-10.1016/j.ijpharm.2020.119492. 10.1016/j.ijpharm.2020.119492 32505579

[83] Jinno J. Kamada N. Miyake M. Yamada K. Mukai T. Odomi M. Toguchi H. Liversidge G.G. Higaki K. Kimura T.. Effect of particle size reduction on dissolution and oral absorption of a poorly water-soluble drug, cilostazol, in beagle dogs. Journal of Controlled Release 111 (2006) 56-64. https://-doi.org/10.1016/j.jconrel.2005.11.013. 10.1016/j.jconrel.2005.11.013 16410029

[84] Koziolek M. Grimm M. Schneider F. Jedamzik P. Sager M. Kühn J.P. Siegmund W. Weitschies W.. Navigating the human gastrointestinal tract for oral drug delivery: Uncharted waters and new frontiers. Advanced Drug Delivery Reviews 101 (2016) 75-88. https://doi.org/10.1016/-j.addr.2016.03.009. 10.1016/-j.addr.2016.03.009 27037063

[85] Vertzoni M. Augustijns P. Grimm M. Koziolek M. Lemmens G. Parrott N. Pentafragka C. Reppas C. Rubbens J. Van Den Αbeele J. Vanuytsel T. Weitschies W. Wilson C.G.. Impact of regional differences along the gastrointestinal tract of healthy adults on oral drug absorption: An UNGAP review. European Journal of Pharmaceutical Sciences 134 (2019) 153-175. https://doi.org/10.1016/-j.ejps.2019.04.013. 10.1016/-j.ejps.2019.04.013 30991092

[86] Vinarov Z. Abdallah M. Agundez J.A.G. Allegaert K. Basit A.W. Braeckmans M. Ceulemans J. Corsetti M. Griffin B.T. Grimm M. Keszthelyi D. Koziolek M. Madla C.M. Matthys C. McCoubrey L.E. Mitra A. Reppas C. Stappaerts J. Steenackers N. Trevaskis N.L. Vanuytsel T. Vertzoni M. Weitschies W. Wilson C. Augustijns P.. Impact of gastrointestinal tract variability on oral drug absorption and pharmacokinetics: An UNGAP review. European Journal of Pharmaceutical Sciences 162 (2021) 105812. https://doi.org/10.1016/j.ejps.2021.105812. 10.1016/j.ejps.2021.105812 33753215

[87] Brouwers J. Brewster M.E. Augustijns P.. Supersaturating drug delivery systems: The answer to solubility-limited oral bioavailability? Journal of Pharmaceutical Sciences 98 (2009) 2549-2572. https://doi.org/10.1002/jps.21650. 10.1002/jps.21650 19373886

[88] Taylor L.S. Zhang G.G.Z.. Physical chemistry of supersaturated solutions and implications for oral absorption. Advanced Drug Delivery Reviews 101 (2016) 122-142. https://doi.org/10.1016/-j.addr.2016.03.006. 10.1016/-j.addr.2016.03.006 27013254

[89] Boyd B.J. Bergström C.A.S. Vinarov Z. Kuentz M. Brouwers J. Augustijns P. Brandl M. Bernkop-Schnürch A. Shrestha N. Préat V. Müllertz A. Bauer-Brandl A. Jannin Vi.. Successful oral delivery of poorly water-soluble drugs both depends on the intraluminal behavior of drugs and of appropriate advanced drug delivery systems. European Journal of Pharmaceutical Sciences 137 (2019) 104967. https://doi.org/10.1016/j.ejps.2019.104967. 10.1016/j.ejps.2019.104967 31252052

[90] Vinarov Z. Abrahamsson B. Artursson P. Batchelor H. Berben P. Bernkop-Schnürch A. Butler J. Ceulemans J. Davies N. Dupont D. Eide Flaten G. Fotaki N. Griffin B.T. Jannin V. Keemink J. Kesisoglou F. Koziolek M. Kuentz M. Mackie A. Meléndez-Martínez A.J. McAllister M. Müllertz A. O'Driscoll C.M. Parrott N. Paszkowska J. Pavek P. Porter C.J.H. Reppas C. Stillhart C. Sugano K. Toader E. Valentová K. Vertzoni M. De Wildt S.N. Wilson C.G. Augustijns P.. Current challenges and future perspectives in oral absorption research: An opinion of the UNGAP network. Advanced Drug Delivery Reviews 171 (2021) 289-331. https://doi.org/10.1016/j.addr.2021.02.001. 10.1016/j.addr.2021.02.001 33610694

